# Plant Genetic Engineering: Technological Pathways, Application Scenarios, and Future Directions

**DOI:** 10.1002/advs.202521040

**Published:** 2026-04-27

**Authors:** Peilin Wang, Wenzhi Wang, Dongjiao Wang, Qibin Wu, Youxiong Que

**Affiliations:** ^1^ State Key Laboratory of Tropical Crop Breeding Institute of Tropical Bioscience and Biotechnology Sanya Research Institute Chinese Academy of Tropical Agricultural Sciences Sanya Hainan China; ^2^ Key Laboratory of Sugarcane Biology and Genetic Breeding Ministry of Agriculture and Rural Affairs National Engineering Research Center for Sugarcane College of Agriculture Fujian Agriculture and Forestry University Fuzhou China

**Keywords:** genetic engineering, plant architecture, quality, stress resistance, yield

## Abstract

As for sustainable food security, plant genetic engineering has emerged as a transformative technology offering innovative solutions. This review comprehensively examines recent advances in plant genetic engineering, from technical foundations to technological innovations, and to multifaceted applications. They transcend the constraints of traditional breeding, including its long cycle and narrow genetic base, showing remarkable potential in crop improvement. By modulating key genes governing plant height, branching, leaf morphology, and root structure, plant architecture can be optimized to enhance light utilization and lodging resistance. Targeted manipulation of genes related to disease and pest resistance, and tolerance to drought, salinity, and temperature extremes, substantially improves resilience to biotic and abiotic stresses. Additionally, by fine‐tuning yield determinants and by engineering photosynthetic pathways, yield potential can be effectively increased. Beyond productivity, genetic engineering facilitates nutritional fortification, improved sensory quality, and enhanced processing characteristics, paving the way for novel crop varieties that integrate nutrition with palatability. Looking forward, coordinated multi‐gene editing, utilization of wild germplasm, strengthened field adaptability testing, and exploration of controllable epigenetic regulation represent key directions for the future. Collectively, these advances will drive plant genetic engineering toward greater precision, efficiency, and intelligence, providing a robust foundation for sustainable agricultural development.

## Graphical abstract

This review maps the fast‐evolving landscape of plant genetic engineering, linking enabling platforms with trait‐focused applications in architecture optimization, stress resilience, yield improvement, and quality enhancement. It highlights how genome editing, transgenic strategies, and emerging multi‐gene approaches reshape breeding pipelines, while outlining technical bottlenecks, regulatory considerations, and future directions. The synthesis offers a practical reference for researchers and students.

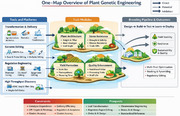

## 1 Introduction

Global agricultural systems are under escalating strain due to population expansion, climate volatility, and resource depletion. By 2050, the global population is expected to exceed 9 billion, driving a steep rise in food demand. Simultaneously, climate change has disrupted agricultural cycles, with increasing incidences of extreme weather events, such as droughts, heatwaves, and heavy rainfall, causing yield losses and crop failures [1, 2]. Water scarcity has intensified, limiting irrigation capacity and forcing reductions in agricultural water use [3, 4]. Meanwhile, urbanization and land degradation have reduced arable land availability, aggravating the pressure on traditional farming systems. These intertwined challenges underscore the urgent need for innovative technologies to ensure global food security.

Since the first successful production of transgenic tobacco in 1983 [5], plant genetic engineering has rapidly evolved, opening a new era in crop improvement. Milestones include early *Agrobacterium*‐mediated transformation and biolistics enabling random integration in the 1980s‐1990s, commercialization of transgenic crops in the mid‐1990s, and the subsequent transition toward site‐specific genome editing. Since 2013, the adoption of CRISPR/Cas systems in plants has markedly improved targeting flexibility and throughput, accelerating both functional genomics and crop trait engineering [6]. Early methods, primarily Agrobacterium‐mediated transformation and particle bombardment, achieved random foreign gene integration with limited efficiency and stability [7]. Advances in molecular biology, gene cloning, and vector design, culminating in the development of precise editing tools such as CRISPR/Cas9, have revolutionized the field, enabling the targeted modification of specific genes for desired traits. Conventional breeding, reliant on natural variation and hybrid recombination, suffers from key limitations: (1) a restricted genetic base confined to intra‐species variation, preventing the transfer of beneficial traits across phylogenetic barriers; (2) low improvement efficiency due to long breeding cycles and undesirable trait linkages, with stable variety development often requiring over a decade; and (3) limited predictability, as specific genes underlying improved traits are often unidentified [8, 9]. These constraints can be interpreted via the breeder's equation, where genetic gain is proportional to selection intensity and accuracy, and to available genetic variance, but inversely proportional to generation interval. Conventional breeding is often limited by restricted usable genetic variance within crossing pools, which reduced selection accuracy for complex traits under strong genotype‐by‐environment effects and long generation intervals that slow cumulative gain. By enabling targeted allele creation and rapid stacking of favorable variants, genetic engineering can increase selection accuracy and shorten the effective improvement cycle. By contrast, plant genetic engineering provides unprecedented advantages for modern crop improvement. First, it offers high precision, enabling direct gene targeting‐for example, CRISPR‐mediated modification of the *GS3* gene in rice precisely regulates grain weight for yield enhancement [10]. Second, it significantly shortens breeding cycles, achieving stable trait expression within 2‐3 years. Moreover, it transcends species barriers, permitting gene transfer from microbes, animals, or even humans‐for instance, insertion of the Cry gene from Bacillus thuringiensis into cotton confers insect resistance unattainable through conventional breeding [11, 12]. Finally, multi‐gene transformation enables simultaneous optimization of yield, stress tolerance, and quality traits, addressing the long‐standing challenge of balancing multiple agronomic characteristics [13].

Plant genetic engineering thus plays a vital role in ensuring food security and enhancing crop adaptability in the face of global challenges. For yield improvement, editing genes such as GW2 in rice can increase yield by 10%–20%, directly contributing to global food supply stability [14, 15]. For stress tolerance, introducing drought‐responsive genes like DREB into wheat and maize improves survival rates under water‐deficit conditions by over 40% [16, 17]. Cross‐environment adaptability has also been achieved: insect‐resistant cotton expressing Cry1Ac reduces pesticide dependence while maintaining stable yields in pest‐prone regions, and expression of CrSMT from Chlamydomonas reinhardtii enhances salinity tolerance [18, 19, 20]. Similarly, herbicide‐resistant crops facilitate conservation tillage, preserving soil structure and improving production efficiency [21]. Moreover, nutritionally enhanced varieties, such as Golden Rice enriched with β‐carotene biosynthesis genes, address micronutrient deficiencies in vulnerable populations, simultaneously improving food quality and health outcomes [22, 23].

In this review, we systematically examine the technical underpinnings and recent advances in plant genetic engineering, focusing on innovations in plant architecture optimization, stress resistance, yield enhancement, and quality improvement. We further discuss emerging trends, challenges, and future directions in the field. This synthesis aims to provide a theoretical and practical framework for the targeted improvement of agronomic traits, cross‐species gene integration, and coordinated enhancement of yield, stress tolerance, and quality, thereby accelerating the transition toward more resilient and sustainable agriculture. The review is therefore structured in a trait‐oriented manner. We begin with a concise overview of enabling platforms, including plant transformation and genome‐editing toolkits, and then discuss major application scenarios in crop improvement, covering plant architecture optimization, stress resistance, yield enhancement, and quality improvement.

## 2 Technical Foundations for Plant Genetic Engineering

**FIGURE 1:**
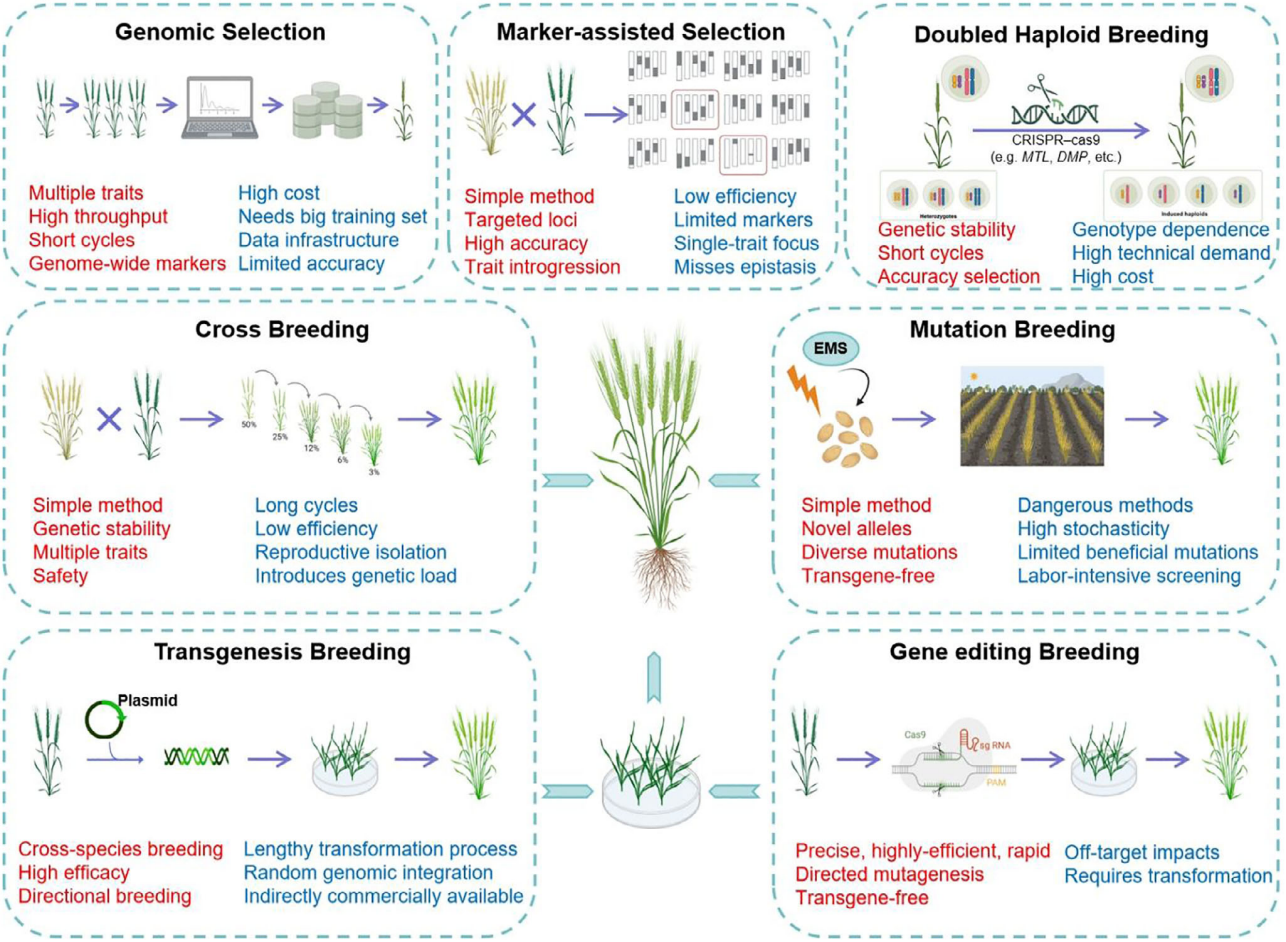
Comparative advantages and limitations of four major breeding strategies: cross‐breeding, transgenesis, mutation breeding, and gene editing. Genomic selection (GS) predicts Genomic estimate breeding value(GEBV)s using genome‐wide markers to accelerate selection for complex traits, but requires a well‐phenotyped training population and substantial data infrastructure(Original breed‐Breeding population‐Prediction models‐GEBVs‐Selection based on GEBV). Marker‐assisted selection (MAS) uses validated markers linked to target loci to improve selection efficiency, but is limited for highly polygenic traits. Doubled haploid breeding (DHB) rapidly produces homozygous lines to shorten breeding cycles, but is genotype‐dependent and technically demanding. Cross‐breeding is simple and produces genetically stable offspring, but is constrained by long breeding cycles and reproductive barriers(percent donor genome expected after each backcross generation). Transgenesis allows cross‐species gene transfer and targeted trait improvement, yet suffers from random gene integration and lengthy transformation procedures. Mutation breeding generates novel alleles without introducing foreign DNA; however, it demands extensive screening and carries potential biosafety risks. Gene editing enables precise, efficient, and transgene‐free genetic modifications, though challenges persist in minimizing off‐target effects and improving transformation efficiency. Image elements are from BioRender (https://www.biorender.com).

Conventional breeding, a classical approach for crop genetic improvement, utilizes naturally occurring genetic variation through systematic hybridization and phenotypic selection. However, this method is constrained by its limited genetic background and protracted breeding cycle. Although mutagenesis breeding can expand genetic diversity during this process, the induced variations are excessively random. In contrast, genetic engineering breeding represents a technological breakthrough by overcoming reproductive barriers, enabling the direct and precise introduction of specific functional genes into target crops. This approach significantly enhances breeding efficiency and precision, and facilitates the creation of novel traits that cannot be achieved through natural hybridization (Figure 1). In practice, conventional breeding is frequently integrated with doubled haploid production, marker‐assisted selection, and genomic selection to accelerate genetic fixation and to improve selection accuracy, although these approaches remain constrained by available variation and multi‐season field evaluation. Plant genetic transformation serves as the core technology of plant genetic engineering, allowing the introduction, stable integration, and expression of foreign genes in plant cells. This capability enables functional characterization of genes, thereby contributing to the elucidation of physiological mechanisms in plants. Furthermore, it facilitates targeted improvement of crop traits‐such as the development of insect‐resistant, disease‐resistant, and high‐yielding varieties‐overcoming the limitations of conventional breeding and providing crucial technical support for agricultural advancement and plant science research [7]. Based on the dependency on vectors, these methods can be categorized into vector‐mediated transformation and direct DNA delivery methods.

### 2.1 Gene Delivery and Transformation Platforms

Vector‐mediated transformation relies on biological vectors to transfer genetic material, with *Agrobacterium*‐mediated transformation being the most prominent and widely adopted method [24, 25]. Optimization of Ti plasmid‐based vectors has significantly improved gene integration efficiency and transgene expression stability [26, 27]. The establishment of transient expression systems now allows rapid gene function validation. Moreover, the method's applicability has expanded from dicots to monocots through the pre‐treatment of callus tissue and the use of hypervirulent Agrobacterium strains, overcoming the long‐standing challenge of monocot transformation [28, 29]. This technology has facilitated the development of numerous transgenic crops: insect‐resistant cotton carrying Bt genes [30], herbicide‐resistant cotton and soybean expressing EPSPS genes [21, 31], bacterial blight‐resistant rice through integration of the *Xa21* gene [32, 33], and improved fruits and vegetables with enhanced storage life and disease resistance, such as tomato and banana [34, 35].

Virus‐mediated transformation employs plant viruses as carriers to deliver foreign genes, capitalizing on their natural infectivity and replication capacity. Once introduced into host cells, viral vectors replicate and express the inserted genes efficiently, supporting both transient expression and potential stable integration [36]. Commonly used viral vectors include single‐stranded RNA viruses such as Tobacco mosaic virus, Sugarcane mosaic virus (SCMV), and Potato virus X, as well as DNA viruses such as Cauliflower mosaic virus [37, 38, 39]. Compared to *Agrobacterium*‐mediated systems, virus‐mediated transformation offers markedly higher efficiency, particularly benefiting plant species that are recalcitrant to tissue culture. Gene delivery can occur through simple mechanical inoculation or insect transmission, eliminating the need for in vitro regeneration. Consequently, viral vectors have found extensive applications in rapid gene functional analysis, molecular farming, and crop trait modification.

Direct transformation methods, in contrast, introduce foreign DNA into plant cells via physical or chemical means, bypassing biological vectors entirely. These techniques are particularly advantageous for species with low Agrobacterium susceptibility, including many monocots and certain dicots [7]. Physical approaches include particle bombardment, electroporation, and microinjection. The gene gun method accelerates DNA‐coated metal microparticles using high‐pressure gas to penetrate plant cell walls and membranes, making it effective across various tissues such as calli and embryoids, and widely applied in crops like maize and wheat. However, this technique entails high costs and a tendency for multi‐copy insertions [40]. Electroporation uses high‐voltage pulses to create transient membrane pores for DNA uptake‐efficient in protoplasts but often stressful to cells [41, 42]. Microinjection, though precise, is technically demanding and labor‐intensive, requiring direct DNA delivery into individual cells or protoplasts under microscopic control [43, 44]. Among chemical methods, polyethylene glycol (PEG)‐mediated transformation is the most widely used. Temporarily increasing membrane permeability in protoplasts facilitates DNA uptake with the advantages of low cost and operational simplicity. However, its efficiency is strongly influenced by cell condition and remains limited to protoplast systems [45]. Overall, direct transformation methods eliminate vector construction and enable rapid gene delivery, making them invaluable for transient expression studies and functional genomics. Nevertheless, challenges such as cellular damage and inconsistent efficiency remain, necessitating integration with complementary techniques to optimize outcomes.​

To facilitate method selection, these delivery platforms can be compared across several practical dimensions, including host range, regeneration dependency, transgene copy number and rearrangement risk, payload capacity, turnaround time, and regulatory considerations. In general, *Agrobacterium*‐mediated transformation often yields relatively cleaner integration patterns and is cost‐effective for many dicots and an increasing number of monocots with optimized protocols, whereas biolistics offers broad host range and genotype flexibility but is more prone to multi‐copy insertions and higher equipment costs. Interestingly, viral vectors enable rapid, high‐level transient expression and are particularly useful for functional screening and molecular farming, but their cargo size, host range, and stability constraints may limit those applications requiring stable inheritance.

### 2.2 Genome‐Editing Toolkits and Variants

Genome editing enables targeted modifications at predefined loci, largely overcoming the randomness of transgene insertion. In plants, CRISPR‐based editors have become dominant because they are programmable via guide RNAs and can be delivered as DNA, RNA, or ribonucleoprotein (RNP) complexes. The major decision points in practice include the choice of nuclease (e.g., Cas9, Cas12a and related enzymes), the repair pathway to be leveraged (NHEJ for knockouts versus HDR or engineered approaches for precise changes), and the delivery/regeneration route used to recover edited events [46].

Different CRISPR modalities are suited to different trait‐engineering goals. Nuclease‐mediated double‐strand breaks are efficient for gene knockouts and regulatory‐element deletions, but can introduce indels and, in multiplex settings, may increase the risk of large deletions or rearrangements. Base editors enable precise single‐nucleotide substitutions without double‐strand breaks, making them well‐suited for generating weak/quantitative alleles and for fine‐tuning enzyme activity or transcription‐factor binding sites, although their editing window and bystander edits require careful design. Prime editing supports a broader spectrum of precise edits, including small insertions, deletions, and replacements, but currently shows variable efficiency across crops and often needs extensive optimization [46]. In addition, CRISPR activation/interference (CRISPRa/i) and epigenome editors provide non‐permanent, regulatory‐layer control for traits where dosage and spatiotemporal expression are critical.

As reported, the suitability of each editing strategy also depends on crop species and trait architecture. For traits controlled by loss‐of‐function alleles, nuclease editing coupled with efficient transformation/regeneration is often sufficient. For elite backgrounds where transgene‐free products are preferred, transient expression or RNP delivery can reduce foreign‐DNA footprint. For complex quantitative traits, multiplex editing, promoter editing, or combinatorial base/prime editing may be more appropriate, but these approaches increase design complexity and require iterative phenotyping to manage pleiotropy and trade‐offs.

## 3 Genetic Engineering for Optimizing Plant Architecture

**FIGURE 2:**
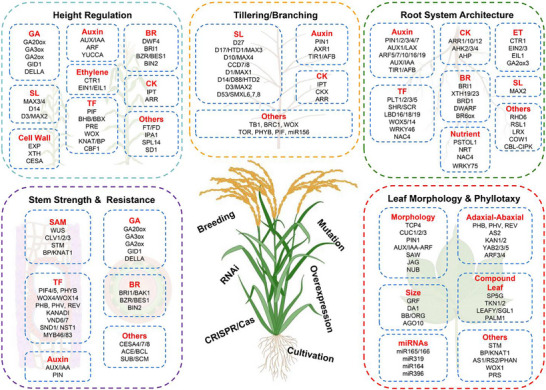
Regulation of plant architecture. Plant architecture improvement encompasses five major aspects: plant height regulation, tillering/branching characteristics, leaf morphology and phyllotaxis, root system architecture, and stem strength and resistance. By identifying and functionally characterizing key regulatory genes within each category, and integrating genetic engineering tools (e.g., RNAi, CRISPR) with traditional breeding techniques (e.g., mutagenesis, cultivation selection), ideal plant architectures can be rationally designed to enhance yield potential and adaptability. Image elements are from BioRender (https://www.biorender.com).

Plant architecture‐the morphological framework formed through long‐term evolution‐fundamentally determines the plant ecological adaptability and agricultural productivity. Understanding and improving crop architecture are critical, as structural traits profoundly influence plant growth dynamics and yield potential [47]. Architectural parameters such as leaf angle, leaf area index (LAI), and plant height collectively regulate light interception and photosynthetic efficiency. Optimized plant height and branching angle enable higher planting densities by allowing more individuals per unit area while preventing canopy overlap that impedes ventilation and light penetration [48, 49]. In addition, compact architecture and sturdy stems enhance lodging resistance by lowering the center of gravity, thereby reducing susceptibility to wind and rain damage and ensuring stable assimilate transport and grain filling [50, 51]. With the rapid progress of genetic engineering, numerous key regulators of plant architecture have been identified (Figure 2), offering novel molecular targets for the rational design of ideal plant ideotypes with enhanced productivity and resilience. To move beyond gene‐by‐gene listing, we organize evidence around three practical questions: (i) beyond controlled environments (e.g., breeding pipelines or field performance), which architectural interventions have been validated? (ii) what exactly was modified (coding sequence vs. promoter/cis‐element vs. specific nucleotide/allele), and which engineering approach was used (transgenesis, RNAi, CRISPR nuclease/base/prime editing)? (iii) what kind of trade‐offs and pleiotropic effects (e.g., yield component penalties, stress sensitivity, root‐shoot coordination costs) have been reported, and how can they be mitigated (e.g., promoter tuning, tissue‐specific/inducible expression, allelic series)?

### 3.1 Regulation of Plant Height

The concept of the “ideal plant type” emerged from selective breeding efforts aimed at optimizing morphological traits for high yield. It is exemplified by the deployment of semi‐dwarf genes during the “Green Revolution” [52]. That is, the introduction of the semi‐dwarf gene sd1 in rice and Rht genes in wheat effectively reduced plant height, minimizing lodging risk while redirecting assimilates from stem elongation to grain development. These genetic innovations facilitated denser planting and significantly boosted global grain yields [53, 54, 55]. Such advancements highlight the importance of coordinated regulation of the “source‐sink‐flow” relationship in achieving ideal plant architecture [56, 57, 58].

Plant height regulation involves both non‐hormonal and hormone‐related genes. Non‐hormonal regulators include genes affecting cytoskeletal organization, cell division, or lignin biosynthesis. In rice, *OsSSD2*, encoding a TRM family protein, is highly expressed in stem internodes and strongly influences plant height: its overexpression leads to shorter plants, whereas CRISPR/Cas9‐mediated knockout results in taller phenotypes, likely through effects on cytoskeletal dynamics or cell proliferation [59]. In soybean, PH13, located on chromosome 13, acts as a major stem elongation promoter by facilitating the degradation of STF1/2 transcription factors. The naturally occurring PH13‐H3 haplotype encodes a partial loss‐of‐function variant with reduced interaction with STF1/2, resulting in STF1/2 accumulation and shorter stems [60]. This haplotype has been widely incorporated into northern soybean breeding programs to minimize elongation and lodging under long‐day conditions. In cotton, silencing *UBP15* and *CUL1* reduces plant height [61], while overexpression of the transcription factor *CBF1* produces a dwarf phenotype with altered architecture [51].

Hormone‐related genes exert dominant control over stem elongation through pathways regulating cell expansion, division, and hormonal signaling. Gibberellin (GA) biosynthesis and signaling genes are particularly critical. In rice, sd1 encodes GA20 oxidase; its mutation reduces bioactive GA synthesis, producing a dwarf phenotype [62, 63, 64]. Similarly, in Arabidopsis, GA1 mutants exhibit severe dwarfism due to disrupted GA biosynthesis [65, 66]. In sugarcane, overexpression of *ScGA20ox*‐introduced into the cultivar GT42 via biolistic transformation‐significantly increased plant height and growth rate while enhancing hormonal metabolism and photosynthetic performance, demonstrating its potential for yield improvement [67]. Brassinosteroids (BRs) also play a pivotal role in stem elongation. The rice DWF4 gene encodes a key enzyme in BR biosynthesis, and reduced expression leads to stunted growth [68, 69]. Similarly, mutation of *BRI1*, which encodes a BR receptor, results in dwarf, thick‐stemmed plants [70, 71, 72]. In addition, auxin transport genes, particularly members of the *PIN* family, regulate plant height by modulating polar auxin flow; altered expression can drastically change elongation patterns. Modern genetic engineering enables precise control of these regulatory networks. Overexpression of GA2ox, a GA catabolic enzyme, decreases GA content and shortens plant stature [73], whereas Rht gene knockout, removing repression of GA signaling in wheat, increases plant height [74, 75]. Collectively, these genetic strategies provide robust molecular tools for fine‐tuning the balance between lodging resistance and light capture, advancing the development of optimized plant architectures.

Height‐reducing alleles can improve lodging resistance and allow dense planting, but excessive dwarfing may reduce biomass, sink size, or stress competitiveness. Therefore, field performance should be interpreted through yield components (panicle/ear number, grain weight) and across environments. Where pleiotropic penalties emerge, quantitative tuning (promoter editing, weak alleles) or tissue‐specific regulation can preserve benefits while avoiding over‐constraint of growth.

### 3.2 Regulation of Branching and Tillering

Branching, or tillering in monocots, is a critical determinant of crop architecture and yield potential. In Arabidopsis thaliana, MAX family genes serve as central regulators of shoot branching [76, 77, 78]. MAX proteins participate in the biosynthesis and signaling of strigolactones (SLs)‐a class of hormones that suppress axillary bud outgrowth, thereby controlling branching patterns [79]. Mutations in MAX genes disrupt SL synthesis or perception, leading to excessive branching, a phenotype demonstrating the negative regulatory role of SL signaling in shoot architecture [80, 81]. Strigolactones are not solely branching suppressors, and they also function as root‐derived signals that shape root system architecture and mediate symbiosis with arbuscular mycorrhizal fungi (AMF). This pleiotropy implies that manipulating SL biosynthesis or perception for shoot architecture can reprogram belowground interactions, with downstream consequences for phosphorus/nitrogen acquisition and disease susceptibility/resistance [82, 83]. Therefore, SL‐pathway engineering should be evaluated using integrated phenotypes that include root traits and symbiotic performance, and dosage‐tuning strategies (e.g., promoter editing or tissue‐specific regulation) may reduce unwanted penalties.

In rice, tillering is a principal agronomic trait directly influencing yield. The *MOC1* gene, encoding a GRAS family transcriptional regulator, is indispensable for axillary meristem formation and tiller bud development [84]. moc1 mutants fail to produce tillers, forming only a single main culm, confirming MOC1 as a positive regulator of tillering [85, 86]. The F‐box protein RCN127 enhances tillering by targeting TCP transcription factors (OsTB1 and OsTCP19) for degradation via the ubiquitin‐proteasome pathway, thereby relieving repression of tiller outgrowth and increasing grain yield [87]. Recent findings also indicate that rhizosphere microbiota influence tiller formation. Screening of 182 rice varieties revealed specific bacterial genera‐*Roseateles* R780 promoting and *Exiguobacterium* R2567 inhibiting tillering‐acting through modulation of the SL signaling pathway [88]. In cucumber, the CsphyB‐CsPIF4‐CsBRC1 regulatory module governs lateral branching by modulating ABA biosynthesis, while mutation of CsphyB reduces axillary bud elongation, highlighting an avenue for light‐ and hormone‐mediated control of branching [89].

The advent of genome editing has further enabled precise manipulation of branching and tillering traits. In wheat, CRISPR/Cas9‐mediated editing of MOC1 homologs has successfully increased productive tiller numbers without compromising other agronomic traits [90]. Similarly, *CRISPR*‐induced knockout of *BnaBRC1* in *Brassica napus* produced double‐copy mutants exhibiting a highly branched phenotype, while disruption of *PvTCP19/22* in *Panicum virgatum* markedly increased tiller number and biomass yield [91, 92]. Together, these studies demonstrate that targeted manipulation of key regulatory genes governing branching and tillering offers an effective strategy for architectural optimization and yield enhancement. The integration of molecular design and gene‐editing technologies provides a powerful framework for developing high‐performance crop ideotypes tailored for specific cultivation environments.

### 3.3 Regulation of Leaf Morphology and Phyllotaxis

Leaf morphology and phyllotaxis are key determinants of plant adaptation and resource utilization, critically shaping photosynthetic performance and overall productivity. Leaf area governs light interception capacity, while leaf angle influences canopy light distribution. Moreover, optimal phyllotactic patterns minimize mutual shading among leaves, collectively maximizing photosynthetic efficiency, assimilate partitioning, and stress tolerance. Consequently, these traits constitute central targets for breeding high‐efficiency plant architectures and improving crop yield and quality.

Leaf morphology and phyllotaxis are controlled by genetic networks that coordinate developmental and hormonal signals. In rice, the LG1 (LIGULELESS 1) gene is a major regulator of ligule development and leaf angle [93, 94]. Mutations in LG1 lead to ligule loss and altered leaf‐sheath inclination. LG1 modulates leaf inclination by influencing cytokinin‐related gene expression, thereby regulating cell elongation and differentiation in the pulvinus [60, 95]. Similarly, *TILLER ANGLE CONTROL 1* (*TAC1*) encodes a membrane‐associated protein that determines leaf angle by controlling asymmetric pulvinus cell growth via auxin distribution, mediated by altered auxin transporter expression [96, 97]. The NARROW LEAF 1 (NAL1) gene is essential for lateral leaf growth, as nal1 mutants exhibit reduced vein number and narrower leaves. NAL1 encodes a plant‐specific protein that regulates vascular bundle patterning through modulation of polar auxin transport, thereby promoting leaf expansion [98, 99, 100]. In Populus tomentosa, natural variation in the YABBY11 gene contributes to differences in leaf shape; a premature stop codon increases marginal serration and leaf area, thereby enhancing photosynthetic efficiency [101]. Designing erect leaf architectures has become a major research direction for achieving optimal canopy light capture and high‐density planting compatibility. Erect leaves reduce mutual shading and improve canopy‐level photosynthetic efficiency. Advances in genome editing have enabled targeted regulation of leaf morphology and phyllotaxis genes to generate ideal erect‐leaf phenotypes. In maize, for example, manipulation of *KNOTTED1‐LIKE HOMEOBOX* (*KNOX1*) family genes allows precise modulation of leaf shape and angle toward more erect orientations [102, 103]. In soybean, targeted editing of leaf morphology‐related genes produced plants with narrower, upward‐oriented leaves, significantly improving light use efficiency and yield under dense planting conditions [104]. In alfalfa, PINNA1, encoding a BELL‐type homeodomain protein, interacts with SINGLE LEAFLET1 (SGL1) to suppress its overexpression and regulate leaflet development. Combined pinna1 and palm1 mutations result in multi‐level compound leaves, elucidating genetic interactions that shape compound leaf complexity [105].

Collectively, these findings establish a theoretical and practical framework for optimizing leaf morphology and phyllotaxis through genetic engineering. The ability to design leaf traits that enhance canopy light interception represents a promising strategy for boosting crop photosynthetic efficiency and yield potential, providing a foundation for future breeding of high‐efficiency plant architectures.

### 3.4 Regulation of Root System Architecture

Root‐system engineering can be contextualized using the ‘steep, cheap and deep’ ideotype, which emphasizes deeper axial rooting, reduced metabolic cost per unit soil exploration, and improved access to deep water and nitrogen [106]. The root system serves as the foundation for plant stability, nutrient acquisition, and environmental adaptability. It absorbs essential resources, including water and mineral nutrients such as nitrogen, phosphorus, and potassium, supporting photosynthesis and metabolic activity [83]. Additionally, roots synthesize signaling molecules (e.g., cytokinins and amino acids) that coordinate shoot growth and respond dynamically to environmental stresses. Under drought, salinity, or nutrient limitation, root systems adapt by modifying morphology‐such as increasing lateral root density‐or through physiological responses like organic acid secretion [107, 108]. Symbiotic interactions with rhizosphere microorganisms, including nitrogen‐fixing bacteria, further enhance nutrient use efficiency. Because root depth and spatial distribution determine water and nutrient uptake efficiency, optimizing root system architecture is central to improving stress resilience and yield stability [109]. Notably, hormonal nodes such as strigolactone signaling link root architectural plasticity with rhizosphere symbioses, highlighting why belowground traits should be co‐evaluated when engineering shoot architecture.

Root system architecture improvement focuses on enhancing root depth, density, and fibrous structure to strengthen adaptability and nutrient uptake. In rice, OsPTR9 encodes an oligopeptide transporter that facilitates both nitrogen uptake and lateral root formation through hormonal regulation. Overexpression of OsPTR9 promotes lateral root primordia formation, increases root density, expands absorptive area, and enhances nitrogen utilization and yield [110, 111]. *OsRBOHE* plays a critical role in root hair development; CRISPR/Cas9 knockout mutants display a 90% reduction in root hair length and 50% reduction in density, confirming its function in cell wall remodeling and root hair elongation [112]. Auxin‐related genes also play major roles in root configuration: OsAUX1, OsAUX3, and OsAUX4 regulate primary root elongation, lateral root initiation, and phosphate starvation response, respectively [113, 114, 115]. OsPIN1b and OsPIN2 modulate root configuration and tiller angle, while OsPIN9 responds to ammonium and regulates tiller growth [116, 117, 118]. In Arabidopsis, ARF12 and PIN1 promote deeper rooting through coordinated auxin signaling [119]. In rice, OsARF12 and OsARF25 control primary root elongation‐mutants show markedly shorter roots [120, 121]‐while OsIAA6, OsIAA9, OsIAA11, and OsIAA13 regulate lateral root initiation [122, 123, 124], and OsIAA23 knockout causes reduced root phenotypes [125]. In wheat, a transposable element (TE) in the promoter of TaVSR1‐B, encoding a vacuolar sorting receptor, is associated with root depth at the booting stage. This TE induces changes in DNA methylation and histone modification, altering TaVSR1‐B expression and root growth. Elevated expression of TaVSR1‐B shortens the elongation zone while extending the maturation zone, promoting deeper root penetration and improved nutrient and water uptake [126]. Additionally, WFZP interacts with TaSYD to regulate root system architecture and nitrogen absorption efficiency [127]. Root responses to environmental stress are governed by complex signaling pathways. Under boron deficiency, the TAF12b/CKH1 factor regulates CEP small‐peptide family genes, altering auxin distribution and remodeling root structure to improve boron‐deficiency tolerance [128]. During crown root development in rice, OsRopGEF10 and OsRAC3 interact with OsAHPs, key cytokinin signaling components. Auxin activates OsRAC3, recruiting OsAHPs to the plasma membrane to suppress cytokinin signaling and promote crown root primordium initiation [129].

Enhancing fibrous root formation is another major breeding goal. Cytokinin pathway genes promote fibrous root initiation and development. In wheat, TaFMO1‐5B, encoding a flavin‐containing monooxygenase, influences root number and length; its modulation optimizes total root length and surface area, improving adaptability to diverse soil environments [130].

### 3.5 Stem Strength and Resistance

Key determinants of crop lodging include plant height, tillering angle, panicle morphology, and stem strength‐the latter primarily defined by stem diameter, wall thickness, and cell wall composition [131, 132]. Plant cell walls, composed mainly of cellulose, hemicellulose, pectin, and lignin, provide structural integrity throughout growth and environmental adaptation [133]. Stem strength serves as a critical factor in conferring lodging resistance, thereby safeguarding yield stability and grain quality. Among the genetic factors influencing this trait, cellulose synthase genes have attracted considerable attention for their central role in maintaining mechanical robustness.

In rice, *OsCesA7* encodes a cellulose synthase catalytic subunit essential for cellulose biosynthesis. Mutations in *OsCesA7* result in brittle culms and dwarfism, with stem cellulose content reduced to approximately 40% of wild‐type levels. The gene is predominantly expressed in mechanical tissues such as vascular bundles and sclerenchyma cells, underscoring its critical function in cellulose synthesis and structural development [134, 135]. During secondary cell wall formation, OsCesA4, OsCesA7, and OsCesA9 assemble into a non‐redundant cellulose synthase complex [136]. Transcriptionally, the NAC29/31‐MYB61 regulatory module activates the expression of secondary wall CESA genes, while DELLA protein SLR1 represses this pathway through direct interaction with NAC29/31. Gibberellin signaling alleviates this inhibition by suppressing the SLR1‐NAC interaction, thereby promoting cellulose biosynthesis [137, 138]. The *STRONG1* gene in rice, encoding a MAP70 family microtubule‐associated protein, negatively regulates stem diameter and lodging resistance. Its knockout significantly increases secondary wall thickness and cellulose deposition, thereby enhancing mechanical strength. *STRONG1* likely modulates secondary wall formation by affecting cortical microtubule stability and orientation. Notably, a natural promoter variation at the M2 site governs haplotype‐specific differences, with the superior allele *Hap‐STRONG1‐C* conferring larger stem diameters and improved lodging resistance, offering a valuable genetic resource for crop improvement [139]. In soybean, the *LRM3* gene‐encoding a U‐box type E3 ubiquitin ligase‐plays a pivotal role in regulating lodging resistance. *LRM3* facilitates degradation of the MYB6 transcription factor through the 26S proteasome pathway. MYB6 directly binds to the promoters of *PAL1* and *PAL2*, key genes encoding phenylalanine ammonia‐lyases that catalyze the rate‐limiting step in lignin biosynthesis, thereby repressing their transcription. By promoting MYB6 degradation, *LRM3* relieves this repression, enhancing lignin accumulation and reinforcing stem strength to improve lodging resistance [140].

## 4 Genetic Engineering for Enhanced Plant Stress Resistance

Pathogens, insect pests, weeds, and abiotic stresses such as drought, salinity, and temperature extremes collectively impose severe constraints on global agricultural productivity and food security. While conventional breeding has contributed significantly to the development of stress‐resistant cultivars, its efficacy remains limited by narrow genetic variation, lengthy selection cycles, and interspecific incompatibility barriers. The advent of molecular biology and genetic engineering has revolutionized crop improvement by enabling the precise and efficient introduction of resistance genes derived from bacteria, viruses, plants, or animals into elite cultivars. This approach confers targeted, durable resistance to specific biotic and abiotic challenges.

**FIGURE 3:**
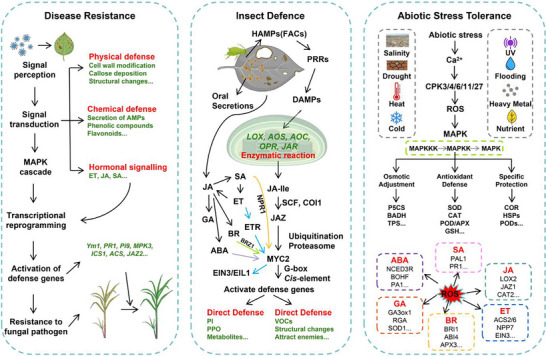
Genetic engineering in plant trait improvement for stress resistance. This model illustrates the key molecular pathways underlying plant responses to various stresses. Disease resistance involves MAPK cascade activation, phytohormone signaling, and physical or chemical defense mechanisms. Insect defense is primarily regulated by JA signaling, leading to both direct defenses (e.g., toxin or metabolite production) and indirect defenses (e.g., volatile‐mediated natural enemy attraction). Abiotic stress tolerance is orchestrated by hormone crosstalk and MAPK cascades, which activate downstream protective genes. Representative core components and genes are highlighted for each resistance mechanism. Arrows indicate regulatory influence and simplified network. Image elements are from BioRender (https://www.biorender.com).

Through transgenic approaches, numerous crops have been engineered to exhibit enhanced resistance to insects, diseases, herbicides, and environmental stresses, demonstrating substantial potential for large‐scale agricultural application. In the following section, we systematically summarize major molecular models and key genes underpinning plant disease resistance, insect resistance, and abiotic stress tolerance (Figure 3). We emphasize their mechanisms of action, successful applications, existing challenges, and broader implications for sustainable agricultural development. All genes discussed in this review are compiled in Supplementary Table S1 with crop species, gene name, engineered trait, functional summary, and corresponding references.

Under laboratory or greenhouse conditions, plants harboring single resistance genes often exhibit nearly complete protection. However, when exposed to natural field environments, resistance frequently diminishes or collapses due to ecological and evolutionary complexities [141, 142, 143]. To avoid overinterpreting single‐gene yield claims, field translation should be evaluated against commonly recommended criteria, including multi‐location and multi‐year testing, appropriate elite genetic backgrounds and agronomic management, robust experimental design and statistics, and assessment of yield components and trade‐offs under realistic production conditions. This “phenotypic attenuation” reflects several key challenges. First, pathogen and pest populations exhibit high genetic variability and rapid adaptive evolution in the field. Generally, laboratory assays employ single or standardized strains, whereas real‐world environments expose crops to continuously evolving pathogen populations. Deploying a single resistance gene exerts strong selection pressure that favors resistance‐breaking variants. Due to pathogen diversification, wheat lines carrying Pm3, which conferred complete resistance to powdery mildew in greenhouse trials, displayed markedly reduced durability under field conditions [144]. To mitigate this, future strategies must prioritize *gene stacking* or *pyramiding* approaches, combining multiple resistance genes or QTLs with distinct recognition spectra using CRISPR or other multigene editing systems. Such pyramided lines, as demonstrated in rice carrying *Piz‐t* and *Pik‐h*, exhibit broader and more durable resistance in the field [145, 146, 147]. Complex abiotic factors in the field‐such as drought, heat, high light intensity, or nutrient limitations‐can modulate plant physiology and compromise immune responses. For example, high temperatures are known to suppress effector‐triggered immunity (ETI) mediated by NLR proteins, leading to “temperature‐sensitive resistance” breakdown [148]. Therefore, engineering durable resistance requires evaluating candidate genes under multifactorial environmental stresses. Identifying alleles with stable performance across variable conditions or engineering NLR proteins for enhanced thermostability represent key directions for future breeding [149]. Integrating resistance traits with “climate‐smart” attributes‐such as drought or heat tolerance‐will be essential for developing resilient cultivars under changing climates. Resistance genes must be optimized alongside agronomic performance. Many highly effective resistance genes impose metabolic costs that reduce growth vigor, yield, or resource efficiency in competitive field environments [150]. Field testing must therefore assess trade‐offs between enhanced defense and key agronomic traits such as yield, quality, and water‐use efficiency. One promising approach is the use of tissue‐specific or inducible promoters‐such as pathogen‐inducible systems‐to ensure resistance gene activation only when needed, minimizing unnecessary energy expenditure and maintaining yield potential [151].

### 4.1 Genetic Engineering for Disease Resistance

Plant diseases, broadly categorized as infectious or non‐infectious, represent one of the most significant threats to global crop production. Infectious diseases caused by fungi, bacteria, viruses, or nematodes often result in extensive yield losses and economic damage. Genetic engineering offers powerful tools for developing disease‐resistant crops by enabling the precise introduction of resistance genes, yielding substantial progress in improving plant immunity. This section summarizes major crop diseases, the key resistance genes identified to date, and their underlying mechanisms (Table 1), providing insight into current strategies and molecular bases for enhancing disease resistance.

**TABLE 1:** Major plants and their related diseases and main resistance genes.

Crops	Disease	Pathogenic bacteria	Resistance Genes	References
Rice	Rice blast	*Magnaporthe oryzae*	*Pb1, Pia, Pib, Pid2, Pid3, Pik, Pik‐h/Pi54, Pik‐m, Pik‐p, Pish, Pit, Pita, Piz‐t, Pi1, Pi2, Pi5, Pi9, pi21, Pi25, Pi36, Pi37, Pi56, Pi63, PiCO39*	[426]
	Bacterial blight	*Xanthomonas oryzae pv. oryzae*	*Xa1, Xa3/Xa26, xa5, Xa7, Xa10, Xa21, Xa23*	[427, 428]
Wheat	Wheat stripe rust	*Puccinia striiformis f. sp. tritici*	*Yr5, Yr10, Yr15, Yr18, Yr36, YrAS2388, Rpi‐amr1*	[429, 430]
	Wheat leaf rust	*Puccinia triticina*	*Lr1, Lr10, Lr21, Lr19/Sr25, Lr24/Sr24, Lr42*	[429, 431]
	Wheat stem rust	*Puccinia graminis f. sp. tritici*	*Sr6, Sr22, Sr31, Sr35, Sr46, AvrSr27*	[432, 433]
	Wheat head blight	*Fusarium graminearum*	*Fhb1, Fhb2, Fhb4, Fhb5, TaFAH*	[434, 435]
Maize	Corn northern leaf blight	*Setosphaeria turcica*	*Ht1, Ht2, Ht3, Htn1*	[436]
	Southern Corn Rust	*Puccinia polysora*	*RppC, RppK, RppP, ABCG11, CCR1, ZmCHit7*	[437, 438, 439]
	Corn head smut	*Sporisorium reilianum*	*ZmWAK*	[440]
Cotton	Verticillium wilt	*Verticillium dahliae*	*GhARM, GhWRKY75, CTL1, GhBON1, GhCBSX3A, GhWRKY41*	[234, 441, 442]
	Fusarium wilt disease	*Fusarium oxysporum f. sp. vasinfectum*	*FOV1, FOV4, FOV7, GhEIN3, GhWRKY48, GhPBL1, GbDP1*/2	[443, 444]
Sugarcane	Mosaic disease	*Sugarcane mosaic virus*	*ScCRT1, ScREM1.5e‐1/‐2, ScUBL5*	[445, 446]
	Sugarcane smut	*Sporisorium scitamineum*	*ScWRKY2, ScDIR*	[175, 447]
Soybean	Soybean cyst nematode	*Heterodera glycines*	*Rhg1, Rhg4, GmUGT88A1*	[448, 449]
	Phytophthora root rot	*Phytophthora sojae*	*NbPrf, GmTAP1, GmPIB1*	[450, 451, 452]
Potato	Potato late blight	*Phytophthora infestans*	*StCDPK16, Rpi‐arm4, R02860, R04373*	[453, 454]
Tomato	Yellow Leaf Curl Virus	*Tomato yellow leaf curl virus*	*Ty‐1, Ty‐2, Ty‐3, Ty‐5*	[455]
	Tomato leaf mold	*Passalora fulva*	*Cf‐2, Cf‐4, Cf‐5, Cf‐12*	[456, 457]
Cucumber	Downy mildew	*Pseudoperonospora cubensis*	*dm‐1, dm‐2, dm‐3, CsRGAs, CsIVP, CsSGR*	[458]
	Powdery mildew	*Podosphaera fusca*	*Csmlo1/8/11*	[459, 460]
Tobacco	Tobacco mosaic virus	*Tobacco mosaic virus*	*Tm‐22, TOM2A, N gene*	[102, 461]

Viral diseases are often regarded as the “cancer of plants,” as even relatively few viral infections can cause devastating crop losses and are notoriously difficult to manage. Most plant viruses attack leaves, leading to chlorosis, necrosis, and deformation, as observed in pepper mosaic, Chinese cabbage stem necrosis, and grapevine fanleaf diseases. Aphid‐transmitted viruses such as Cucumber mosaic virus (CMV), Beet yellows virus (BYV), and Barley yellow dwarf virus (BYDV) are especially prevalent. A well‐established antiviral approach is coat protein (CP)‐mediated resistance [152], where introducing a viral *CP* gene enables the transgenic plant to produce viral coat proteins that confer protection. In tobacco, antiviral resistance was engineered by transgenesis expressing the tobacco mosaic virus (TMV) coat protein (CP), and validated in infection assays under controlled conditions, however without discussion on trade‐offs [153, 154]. Similarly, the incorporation of the Papaya ringspot virus (PRV) CP gene into papaya rendered plants highly resistant to PRV infection under field conditions [154]. In wheat, antiviral resistance was engineered by root‐specific transgenic expression of the NLR gene *Ym1*, which recognizes the WYMV coat protein and triggers localized cell death to block viral invasion and upward spread, and the evidence was only demonstrated in controlled infection/phenotyping assays [155]. RNA interference (RNAi) represents another key antiviral mechanism in plants. Upon infection, double‐stranded viral RNA (dsRNA) is recognized and cleaved by Dicer‐like (DCL) nucleases into small interfering RNAs (siRNAs), which guide Argonaute (AGO)‐mediated cleavage of complementary viral transcripts, thus suppressing viral replication. Engineered hairpin RNA (hpRNA) constructs amplify this process by producing dsRNAs that yield abundant virus‐specific siRNAs. For instance, transgenic potato plants expressing hpRNA targeting Potato virus Y (PVY) sequences displayed markedly reduced viral accumulation and disease incidence [156, 157]. Similarly, transgenic rice line ZJU‐4K, expressing a fused hpRNA targeting conserved regions of four major rice viruses‐Rice black‐streaked dwarf virus (RBSDV), Southern rice black‐streaked dwarf virus (SRBSDV), Rice stripe virus (RSV), and Rice ragged stunt virus (RRSV) [158]‐exhibited broad‐spectrum resistance and near‐immunity to SRBSDV and RSV under artificial inoculation [159].

Bacterial and fungal infections remain among the most destructive plant diseases. The plant innate immune system employs two main defense strategies: Pattern‐Triggered Immunity (PTI) and Effector‐Triggered Immunity (ETI). In PTI, pattern recognition receptors (PRRs) detect conserved microbial signatures. For example, AtFLS2 encodes a receptor that perceives bacterial flagellin and initiates downstream MAPK cascade signaling, leading to reactive oxygen species (ROS) production, callose deposition, and activation of defense genes such as PR1 [160]. ETI, in contrast, relies on intracellular immune receptors encoded by NLR (Nucleotide‐Binding Leucine‐Rich Repeat) genes that recognize pathogen effectors and trigger a strong hypersensitive response. In tomato, the *Pto* gene encodes a protein kinase that recognizes the *Pseudomonas syringae* effector AvrPto, activating ETI and conferring resistance [161].

Plant hormones regulate bacterial defense pathways. The salicylic acid (SA) pathway primarily mediates resistance against biotrophic bacteria, with ICS1 being essential for SA biosynthesis. Upon infection, ICS1 induction elevates SA levels, which activate PR genes (e.g., PR1) to strengthen resistance [162]. In contrast, jasmonic acid (JA) and ethylene (ET) signaling mediate defense against necrotrophic bacteria. Infection by Ralstonia solanacearum in tomato activates LOX (JA synthesis) and ACS (ET synthesis) genes, enhancing resistance via coordinated hormonal signaling [163]. Cross‐regulation among SA, JA, and ET ensures a balanced immune response. CRISPR/Cas9 gene editing now enables targeted improvement of bacterial resistance. Editing SlJAZ2 in tomato enhanced resistance to Pseudomonas without compromising yield [164], while editing *MdDIPM4* in apple improved resistance to *Erwinia amylovora* [165], and disruption of Os8N3 in rice conferred resistance to Xanthomonas oryzae pv. oryzae [166, 167]. Additionally, CRISPR/Cas9 can be applied to bacterial genomes, enabling functional dissection of virulence factors in *Pseudomonas* and *Xanthomonas* species.

Plants deploy both constitutive and inducible physical and chemical defenses as their first barrier against fungal invasion, with numerous genes playing pivotal roles in these processes. Physical Barriers: Genes governing cell wall biosynthesis and modification critically influence plant resistance to fungal pathogens. Among them, cellulose synthase genes (CesA) participate in cellulose production, a major structural component of the cell wall. Upon fungal challenge, CesA expression is upregulated, leading to increased cellulose deposition and lignification. This reinforcement enhances the mechanical strength of the wall, impeding fungal hyphal penetration and conferring structural resilience [168]. Chemical Defenses: Genes responsible for phytoalexin biosynthesis constitute an essential component of chemical defense. Phytoalexins are antimicrobial secondary metabolites synthesized in response to pathogen attack. In grapevine, stilbene synthase (STS) genes are activated upon powdery mildew infection, promoting the accumulation of stilbene phytoalexins that restrict fungal growth and development [169, 170]. In grapevine, resistance to powdery mildew was enhanced by stable transgenic expression of *VqNSTS2* (stilbene synthase). The inoculation assays in both Arabidopsis and susceptible grapevine genotypes revealed that VqNSTS2 localized around haustoria and further restricted spore invasion [171]. Additionally, plants secrete hydrolytic enzymes such as chitinases and β‐1,3‐glucanases, which degrade chitin and β‐1,3‐glucan‐the principal components of fungal cell walls‐further strengthening antifungal defense through enzymatic degradation. Signal transduction in antifungal immunity involves highly coordinated genetic regulatory networks. During both PTI and ETI, genes constituting the mitogen‐activated protein kinase (MAPK) cascade play a crucial role. In rice challenged by *Magnaporthe oryzae*, *OsMPK3*, *OsMPK4*, and *OsMPK6* are rapidly activated, phosphorylating downstream transcription factors that regulate defense‐related gene expression and enhance resistance to blast disease [172]. Transcription factors are central regulators of antifungal gene expression, with the WRKY family being particularly well‐studied. In Arabidopsis thaliana, WRKY46, WRKY54, and WRKY70 are strongly induced upon fungal infection, binding to promoter regions of defense genes to coordinate their transcriptional activation [173]. The “enhancer‐promoter‐transcription factor” regulatory module fine‐tunes immune homeostasis by restricting excess accumulation of the phytoalexin camalexin. In plants, fungal resistance was enhanced by CRISPR/Cas9‐mediated editing of *CPIE35* or *WRKY15*. Notably, resistance increased without constitutive immune activation or growth inhibition, indicating reduced pleiotropic cost [174]. In sugarcane, ScWRKY2 acts as a negative regulator of smut resistance by suppressing ScLRR‐RLK expression. Moreover, ScWRKY2 interacts with the chloroplast protein SCPSbP to induce ROS‐scavenging genes, maintaining ROS homeostasis, and modulating stress signaling [175].

Genetic engineering represents a key strategy for developing cultivars with enhanced fungal resistance. In rice, resistance (*R*) genes such as Pi‐ta and Pi9 encode NLR proteins that recognize specific Magnaporthe oryzae effectors, conferring race‐specific resistance [176]. Further optimization through genome editing has expanded the potential of R gene‐mediated protection. CRISPR/Cas9 knockout of TaMLO in wheat conferred durable, broad‐spectrum resistance to powdery mildew [177], while deletion of *miR482b* and *miR482c* in tomato improved resistance to late blight [178, 179]. In parallel, CRISPR/Cas9 has been successfully applied to edit fungal genomes directly, reducing pathogen virulence. Targeted disruption of key virulence genes in Phaeosphaeria nodorum (wheat glume blotch), Ustilaginoidea virens (rice false smut), Phytophthora sojae, and Phytophthora capsici markedly decreased their pathogenicity [180, 181].

### 4.2 Genetic and Molecular Mechanisms of Insect Resistance

Insect pests exert profound and multifaceted impacts on plant growth, development, reproduction, and ecosystem stability. Direct structural damage occurs when herbivores feed on vegetative organs such as roots, stems, and leaves, often targeting reproductive structures including flowers, fruits, and seeds. Physiological disruption arises as insect feeding inhibits photosynthesis, impairs assimilate transport, and perturbs hormonal balance, weakening overall plant metabolism. Secondary infestations are frequently induced, as insect feeding wounds facilitate pathogen entry, including viruses, fungi, and bacteria. Reduced ecological fitness is also observed; infested plants exhibit lower tolerance to environmental stresses such as drought, high temperature, and chilling, leading to stunted growth, impaired reproduction, and diminished competitiveness. Chronic infestation can result in local population decline and disrupt ecosystem stability [182]. Insect herbivory imposes cascading damage‐direct tissue loss, disrupted photosynthesis and assimilate transport, hormonal imbalance, and wound‐facilitated pathogen entry‐ultimately reducing stress tolerance and ecological fitness [182, 183]. Plants counter this pressure through integrated defenses: physical barriers (cuticle, trichomes, sclerenchyma), chemical deterrents (alkaloids, phenolics, terpenoids, and digestive inhibitors), and ecological/biological strategies [184, 185, 186, 187].

In maize, loss‐of‐function of the cuticular wax gene *ZmGL8* (3‐ketoacyl reductase) was associated with JA pathway activation and stronger anti‐herbivore defense, consistent with insect resistance phenotyping [188]. Rice provides a clear quantitative example: oscerk1 lines show a thinner wax layer and faster planthopper probing/feeding, whereas OsCERK1 overexpression thickens wax and cuts oviposition by ∼50%, as demonstrated in brown planthopper bioassays [189, 190, 191]. Mechanistically, ACL1 modulates wax deposition by competing with HD‐Zip IV factors ROC4/5 to repress wax‐biosynthetic genes (e.g., KCS), linking cuticle traits, bulliform cell development, and combined drought‐insect outcomes [192]. Trichomes, both glandular and non‐glandular, impede insect movement or feeding, sometimes secreting secondary metabolites such as alkaloids and terpenoids. In *Arabidopsis*, the GL1‐GL3‐TTG1 complex activates *GL2* to promote trichome formation. Trichomes show a similar ‘barrier’ logic: *gl1* plants lacking trichomes allow 2‐3× higher aphid feeding, while GL1 overexpression increases trichome density and reduces aphid survival in feeding/survival assays. In rice, mutation of GL1 eliminates leaf and glume trichomes, diminishing aphid resistance [193]. In cotton, *GhMYB212* promotes leaf trichome and fiber development. Its overexpression increases trichome density by 50%, limits cotton bollworm feeding, reduces leaf damage by 40%, and enhances fiber lignification and mechanical resistance [194]. The key gene HIC directly activates GhEXPA2, increasing trichome density and branching in upland cotton, which effectively reduces aphid feeding and damage [195].

Plants deploy sophisticated chemical defenses as a central anti‐herbivore strategy, relying on the synthesis, accumulation, or secretion of specialized secondary metabolites to deter insect feeding. These compounds can directly poison insects, impair growth and development, disrupt digestion, or indirectly enhance plant defense by attracting natural enemies. Chemical defenses depend on both the biosynthesis of secondary metabolites and the regulation of defense‐related signaling pathways. The MIXTA family transcription factor *SlMIXTA*‐like (*SlMYB12*) exemplifies this strategy by regulating type VI glandular trichome development. By overexpressing *SlMIXTA‐like* (SlMYB12), tomato lines doubled type VI glandular trichomes and raised acyl sugars, translating into >50% mortality against whitefly and Colorado potato beetle in bioassays [196, 197]. Similarly, the cytochrome P450 monooxygenase CYP71A1, which converts tryptamine to serotonin, modulates insect resistance in rice. CYP71A1 mutants, with reduced serotonin, showed enhanced resistance to brown planthopper, white‐backed planthopper, and stem borers [198]. Inactivation of the rice *OsSL* (*SEKIGUCHI LESION*) gene, encoding a CYP71A1 protein, elevated salicylic acid (SA) levels and conferred increased resistance to both blast fungus and brown planthopper [199]. Hormone‐mediated signaling underpins many chemical defenses, involving ethylene (ET), abscisic acid (ABA), jasmonic acid (JA), and SA pathways [200, 201, 202]. In tomato, EIN3 activates *SlERF1*, inducing proteinase inhibitor II (PinII) and terpenoid biosynthesis genes, which inhibit cotton bollworm larval growth. In rice, *OsEIL1* promotes JA biosynthesis by degrading the negative regulator *OsSEI1*, enhancing resistance to brown planthopper [203, 204, 205]. ABA‐linked engineering can also be effective, that is, *OsNCED3* overexpression elevates ABA and reduces planthopper oviposition/nymph survival via defense enzyme activation [10]. In tobacco, activating ABA perception through *NtPYL4* overexpression promotes lignin deposition and terpenoid synthesis, thereby limiting budworm feeding [206]. JA‐mediated defenses involve the induction of proteinase inhibitors and metabolites that impair insect digestion. In legumes, JA‐induced *PI‐II* genes inhibit gut proteases, significantly suppressing lepidopteran larval growth [207]. In rice, OsJAZ9 overexpression suppresses JA signaling and reduces planthopper resistance, whereas knockout lines exhibit enhanced JA responses, increased mixed‐linkage β‐1,3;1,4‐glucan (MLG) accumulation, and inhibited feeding [200]. Lipoxygenase (LOX)‐mediated JA biosynthesis activates COI1 receptor signaling and downstream defense genes (e.g., *PinII*, terpenoid biosynthesis), while OsLOX1 overexpression enhances volatile terpenoid release, attracting parasitic wasps [208, 209]. SA signaling predominantly targets piercing‐sucking pests like aphids, mediated via NPR1‐induced pathogenesis‐related (PR) protein expression [210]. Hormonal crosstalk can further refine defense: in cotton, DELLA proteins (GA signaling repressors) interact with JA signaling to modulate resistance [211], while the *Arabidopsis npr1* mutant exhibits hyperactivated JA pathways, decreasing aphid resistance but enhancing defense against chewing insects like diamondback moth [212].

Insect resistance is also mediated through symbiotic interactions and volatile‐mediated recruitment of natural enemies, coordinated by complex genetic networks. Symbiosis with mycorrhizal fungi, endophytes, and rhizobia induces defense gene expression, while synthesis and secretion of specialized compounds repel herbivores or attract predators [187]. In alfalfa, the β‐N‐acetylhexosaminidase gene MtHEXO2 hydrolyzes chitin oligosaccharides, regulating mycorrhizal colonization and the expression of symbiosis‐associated genes like phosphate transporters. Loss of MtHEXO2 compromised mycorrhizal colonization and lowered defense gene expression (e.g., PAL), which coincided with higher susceptibility to root‐knot nematodes in phenotyping assays [213]. Endophytic bacteria also contribute to insect resistance. Endophyte‐derived toxins can substitute for plant‐encoded defenses: sugarcane endophyte Serratia marcescens produces serralysin, which targets insect midgut APN and disrupts larval tissues, with expression induced by herbivory [214, 215]. In Acacia, the acetyltransferase AcAAT4 esterifies (Z)‐3‐hexenol to produce (Z)‐3‐hexenyl acetate, a volatile that repels tea green leafhoppers and attracts natural enemies. AcAAT4 expression is JA‐dependent and directly activated by transcription factor AcMYC2b, forming a “herbivory stress → JA signaling → AcMYC2b → AcAAT4 → volatile release” cascade [216]. Rhizobia‐mediated defense also modulates plant chemical resistance. In rice, overexpression of *OsWRKY36* suppresses defense against brown planthopper, white‐backed planthopper, small brown planthopper, rice blast, and bacterial blight, whereas *OsWRKY36* knockout enhances resistance. *Bradyrhizobium japonicum* induces *PAL* gene expression, stimulating the phenylpropanoid pathway and increasing phenolic compounds (e.g., ferulic acid) and lignin accumulation, strengthening resistance to root‐knot nematodes. Thus, *OsWRKY36* negatively regulates defense by repressing *PAL*, and its knockout substantially boosts insect resistance [217]. Plants achieve insect resistance by synthesizing and releasing diverse volatile compounds, including terpenoids, aldehydes/alcohols, and esters/ketones. Terpenoids, a large class of natural organic compounds classified by their isoprene unit composition, perform multifaceted roles in plant defense and have pharmaceutical relevance. In maize, the terpene synthase gene TPS10 catalyzes the production of sesquiterpenes, such as farnesene and (E)‐α‐bergamotene, which attract parasitic wasps (Cotesia spp.). The transcription factor EREB58 binds to the GCC‐box in the TPS10 promoter, activating expression and establishing a regulatory cascade: “herbivory → EREB58 → TPS10 → volatile release → natural enemy attraction” [218]. In cotton, GhTPS12 catalyzes (3S)‐linalool synthesis, a key volatile that repels cotton bollworm while attracting parasitic wasps [219]. In tomato, the bHLH transcription factor *SlJIG*, downstream of the JA pathway core regulator *MYC2*, modulates TPS gene expression and enhances resistance to cotton bollworm. *SlJIG* knockout reduces terpenoid content, increases leaf attractiveness, and promotes larval feeding [220]. Aldehyde and alcohol volatiles also contribute to defense. In Arabidopsis, the WRKY transcription factor AtWRKY46 activates lipoxygenase (LOX) and aldehyde dehydrogenase (ALDH) genes, promoting trans‐2‐hexenal synthesis and enhancing aphid repellence. Loss of AtWRKY46 reduces trans‐2‐hexenal release by 50% and significantly lowers aphid resistance [221]. In tea (Camellia sinensis), CsTSB2 encodes tryptophan synthase, mediating indole production. Indole functions as a volatile attractant for parasitic wasps targeting the tea geometrid (Ectropis obliqua), and its expression follows a diurnal pattern, peaking during daytime light conditions [222].

Esters and ketones similarly mediate defense. In rose, *rcmcs2l* encodes myristyl alcohol synthase, producing a volatile repellent that inhibits beet armyworm (*Spodoptera exigua*) oviposition. Its expression is induced by powdery mildew infection, and gene transfer into uninfected roses confers insect resistance independent of pathogen presence. In tea, the β‐elemene synthase gene *CsELE* promotes β‐elemene synthesis, which synergizes with the JA signaling pathway. β‐Elemene amplifies JA signaling by activating biosynthetic genes (e.g., *AOS*) and defense genes (e.g., *PDF1.2*), establishing a coordinated defense cascade: “herbivory → CsELE → β‐elemene → JA signal amplification → enhanced insect resistance” [223].

### 4.3 Drought and Salinity Tolerance

Global climate change and environmental degradation have intensified drought and salinization, severely constraining plant growth and agricultural productivity. Currently, approximately 20% of the world's cultivated land is affected by salinity, while 43% faces varying degrees of drought stress [224, 225]. These stresses suppress plant growth and development, leading to substantial yield and quality losses [226]. Conventional breeding for drought and salt tolerance remains limited by long selection cycles, low efficiency, and narrow genetic diversity [227]. In contrast, advances in genetic engineering provide powerful tools for rapidly enhancing stress tolerance by introducing exogenous genes across species barriers.

Plant drought and salinity tolerance can be summarized into three interacting layers: osmotic adjustment, antioxidant protection, and signal transduction/transcriptional control [228]. Osmolytes such as proline, glycine betaine, and sugars help maintain cellular water status; representative control points include P5CS/P5CR for proline accumulation [229, 230], BADH for glycine betaine synthesis [231, 232], and TPS‐mediated trehalose production for improved drought and salinity resilience [233, 234]. At the signaling level, Ca^2+^‐responsive modules contribute to osmotic responses, including CPK3/4/6/11/27 ‐ SnRK2 phosphorylation and stress activation [235], and OsGF14f‐OsbZIP23 co‐regulation in rice [236]. Drought and salinity commonly trigger excessive ROS accumulation, causing oxidative damage unless buffered by enzymatic and non‐enzymatic antioxidant systems [237, 238]. Core enzymes include SOD, CAT, POD, and APX [239], and their enhancement is frequently associated with improved tolerance. For instance, TaSOD2 overexpression increased SOD activity and strengthened salt/oxidative tolerance in wheat (with supporting assays in Arabidopsis) [240]. In maize, drought resilience has been linked to transcriptional activation of antioxidant outputs, such as ZmCPP2 binding the ZmSOD4 promoter via interaction with ZmSK1 [241]. Heterologous expression provides additional validation routes: PtCAT2 overexpression in Arabidopsis elevated CAT activity (∼5×) together with SOD/POD, improving drought tolerance [242]. Beyond single genes, combinatorial activation can amplify downstream defenses‐co‐transfection of ZmEREB180 with ZmMPK1 or ZmMPK3 boosted multiple antioxidant/root‐development genes and enhanced waterlogging tolerance [243]. Non‐enzymatic antioxidants (AsA, GSH, carotenoids) further contribute to redox balance [244, 245, 246].

Perception of drought and saline‐alkali stress initiates complex signal transduction cascades that regulate gene expression to activate stress adaptation [247]. The mitogen‐activated protein kinase (MAPK) cascade is central to these responses, involving sequential phosphorylation of MAPKKK, MAPKK, and MAPK components that transmit signals to downstream effectors [248]. Calcium signaling also plays a pivotal role [248, 249]. Stress‐induced cytosolic Ca^2^^+^ surges act as second messengers, binding calmodulin (CaM) or calcium‐dependent protein kinases (CDPKs) to modulate stress‐responsive targets [250, 251]. Buckwheat provides a clear MAPK module example: stress induces FtMPK3/4/8, and functional perturbation supports causality‐VIGS silencing of FtMPK3 reduced proline and antioxidant enzyme activities, whereas FtMPK3 overexpression lowered MDA (∼40%) and improved photosynthetic efficiency (∼25%) under drought/salinity assays [252]. In cotton, GhMAPKK5 serves as a key regulator of drought and salinity tolerance, functioning through the MEKK3/8/31‐MAPKK5‐MAPK11/23 cascade to modulate WRKY transcription factors and ABA/proline biosynthesis pathways. Overexpression of GhMAPKK5 notably improved germination rate and root development in Arabidopsis [253]. Collectively, engineering successes in this domain tend to converge on water‐status maintenance (osmolytes), ROS buffering (antioxidants), and signal‐module tuning (MAPK/Ca^2+^/ABA).

### 4.4 Extreme Temperature Tolerance

Climate change increasingly exposes crops to temperature extremes, with heat waves, cold snaps, and their associated pest/disease pressures collectively threatening yield stability. Meta‐analyses indicate that each 1°C global warming can reduce wheat, rice, and maize yields by ∼3%–7%, and during rice booting, extreme heat (>38°C) can induce pollen sterility and cut seed set by >50% [254, 255]. Conversely, chilling limits cultivation at high latitudes. In Northeast China, rice can lose ∼10% yield annually due to low spring temperatures [253, 256].

Cold tolerance is frequently organized around the ICE‐CBF‐COR axis: ICE factors activate CBFs, which then induce COR genes that stabilize membranes and reinforce antioxidant capacity [257]. Post‐translational tuning can strengthen this module‐e.g., phosphorylation and SIZ1‐mediated SUMOylation of ICE1 enhances binding to the CBF3 promoter and improves freezing tolerance in Arabidopsis [258]. Beyond this canonical route, regulatory diversity is evident across species: ScDREBA5 is strongly cold‐induced in *Syntrichia caninervis* and promotes freezing tolerance via COR activation [259], whereas in wheat, an AP2‐domain deletion in *TaCBF12* compromises CRT/DRE binding and reduces freezing tolerance, contrasting with cases where higher *TaCBF14*/*15* expression elevates COR14B and DHN5 transcription [260]. Additional entry points include lipid‐homeostasis control (maize HSF21 regulating GPAT/SAD pathways) [261] and growth‐defense balancing via hormone metabolism, as *UGT71A60* overexpression in tea increases antioxidant activity and survival under cold stress. Epigenetic variation can also be causal: in rice, cold stress suppresses *MET1b*, leading to *ACT1* promoter hypomethylation, enabling *Dof1* binding and heritable cold‐response activation that supports northward adaptation [262]. Crop‐relevant cold‐tolerance examples in Brassica also highlight translational routes beyond model‐based ICE‐CBF exemplars. In *Brassica napus*, integrative omics analyses identified a low‐temperature adaptability module in which Bna‐miR397a post‐transcriptionally regulates the laccase gene *BnaLAC2*. Modulating this miR397a‐LAC2 axis alters cell‐wall/lignin remodeling and ROS homeostasis under low temperature, thereby enhancing low‐temperature adaptability. Notably, the authors further supported functional relevance by testing the module's effects on freezing tolerance in Arabidopsis, suggesting a conserved role of miR397‐LAC2 across Cruciferae [263].

Heat stress disrupts photosynthesis and proteostasis, and plants often rely on *HSFs* (notably *HSFA1*/*HSFA2*) to activate HSP chaperones (e.g., *HSP101*/*HSP70*) via HSE motifs [264]. Several interventions demonstrate different engineering logics. In *Populus*, *PtHSFA4a* directly activates *APX1* and *HSP* promoters, strengthening both antioxidant defense and protein folding [265]. In Arabidopsis, boosting the ethylene‐linked branch‐*ERF95*/*ERF97* overexpression‐upregulates heat‐responsive genes (*HSP21*, *HSP17.6A*) and raises survival to ∼80% after 43°C exposure [266]. Translational evidence is even strongest in rice: QT12 (NF‐YB9) mutant lines relieve repression of HSFA1, improving seed set and grain quality under heat, and multi‐site field trials reported up to 92.5% yield gains under heat stress [267]. Regulatory RNAs provide another lever‐heat‐induced *miR165/166* targets PHB, releasing *HSFA1* activity to enhance HSP expression [268, 269]. Hormones further integrate these pathways: ABA promotes antioxidant enzymes and *HSPs* to reduce membrane damage [270, 271], while EIN3 can reinforce this thermotolerance module through *ERF95*/*97* activation [266].

*Thermo‐tolerance 1* (*TT1*), first cloned as a major QTL from African rice, encodes a 26S proteasome α2 subunit. Favorable natural alleles enhance the clearance of heat‐induced denatured proteins and improve thermotolerance, providing a mechanistically grounded target for breeding and engineering. *Thermo‐tolerance 2* (*TT2*) was identified as a natural QTL encoding a G‐protein γ subunit. Loss‐of‐function alleles increase wax retention under heat and improve thermotolerance during both vegetative and reproductive stages without obvious yield penalty. More recently, *Thermo‐tolerance 3* (*TT3*) was resolved as a single‐locus module consisting of two antagonistic genes (*TT3.1/TT3.2*) that protect chloroplast function under heat stress. Near‐isogenic lines carrying the favorable *TT3* haplotype exhibited substantially reduced heat‐induced yield losses, supporting *TT3* as a practical stacking component for reproductive‐stage heat resilience [272, 273].

Despite representing opposing thermal extremes, cold and heat stress responses share extensive molecular and regulatory parallels, facilitating cross‐talk and adaptive coordination. ABA and ethylene participate in both stress responses: ABA enhances cold tolerance via the ICE‐CBF cascade while simultaneously promoting thermotolerance through HSF activation [274]. Likewise, EIN3 modulates both heat‐responsive (ERF95/97) and cold‐responsive (CBF) genes, forming an integrative regulatory framework for temperature adaptation [266]. Epigenetic mechanisms offer another convergence point‐cold stress induces *ACT1* promoter hypomethylation, while heat stress increases histone H3K9 acetylation, both facilitating stress‐related gene expression [262, 275]. Furthermore, both stresses induce ROS accumulation, necessitating strong antioxidant defenses. In maize seedlings, SOD, POD, and CAT activities rise significantly under chilling stress, while *OsAPX1* overexpression in rice enhances tolerance to both cold and heat [276, 277]. Similarly, in cotton, *GhCOX11* interacts with glutathione peroxidase (GPX) family members to cooperatively scavenge ROS, conferring improved tolerance to drought and cold stress [278]. Overall, temperature‐tolerance targets span signaling hubs, transcriptional regulators, and redox modules, but field‐scale validation remains the key filter for prioritizing candidates for breeding pipelines.

### 4.5 Waterlogging/Flooding Tolerance and Herbicide Resistance

Waterlogging and flooding impose hypoxia/anoxia stress that disrupts respiration, causes ROS bursts upon re‐oxygenation, and often triggers severe yield losses in humid or monsoon cropping systems [279]. Engineering strategies for flooding tolerance typically target (i) oxygen sensing and hypoxia signaling, (ii) carbohydrate conservation and anaerobic metabolism, and (iii) root aerenchyma formation and architectural adaptation. Translationally, flooding tolerance has benefited from deploying major‐effect alleles governing escape or quiescence strategies, and combining these with quantitative modules that stabilize growth and recovery under fluctuating water levels. Future work should emphasize multi‐environment field validation across variable flooding regimes to ensure durability and to quantify yield‐component trade‐offs.

Herbicide resistance is among the most widely deployed targets in crop biotechnology because it directly enables effective weed control and supports conservation agriculture. Engineering approaches include (i) target‐site resistance via amino‐acid substitutions that reduce herbicide binding while preserving enzyme function, and (ii) metabolic resistance via enhanced detoxification or degradation pathways. Compared with stress‐tolerance traits, herbicide‐resistance engineering often has clearer deployment pathways and field performance metrics. However, stewardship and integrated weed management are essential to delay resistance evolution in weed populations.

## 5 Plant Genetic Engineering for Yield Improvement

Enhancing crop yield is central to global food security and sustainable development. FAO projections suggest the global population may exceed 9.7 billion by 2050, implying a >50% increase in food production compared with today. Yield gains‐raising productivity per unit area under limited arable land‐remain the most direct lever to narrow the supply‐demand gap and reduce food‐related instability [280, 281, 282].

Yield improvement is a multi‐trait outcome emerging from coordinated optimization of source capacity, sink strength, resource‐use efficiency, and stress resilience, rather than from single‐gene effects alone. In practice, engineering often faces allocation trade‐offs and pleiotropy, where improving one component can penalize others. These risks can be reduced through system‐level design (e.g., modular expression, tissue/cell‐type‐specific or inducible promoters) and by deploying weak or quantitative alleles to tune effect size. Trait stacking is therefore best implemented stepwise with iterative phenotyping of component traits and whole‐plant performance, followed by multi‐environment field validation to avoid “greenhouse winners” that fail under agronomic conditions.

**FIGURE 4:**
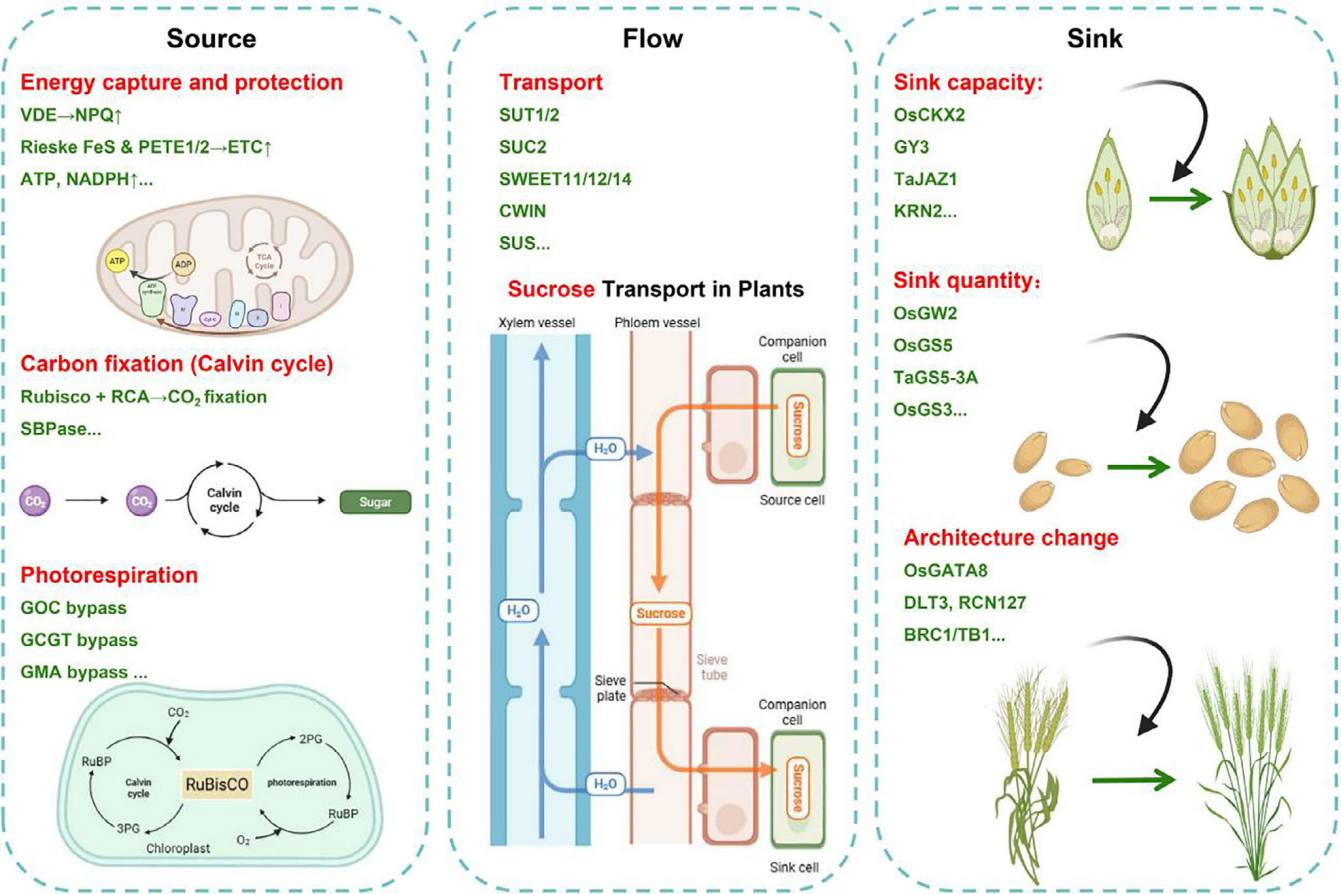
Schematic representation of “Source‐Flow‐Sink” regulation in plants. The diagram outlines key processes from source to sink. The Source section depicts light energy capture and protection (e.g., VDE, ETC), carbon fixation via the Calvin Cycle (e.g., Rubisco, SBPase), and photorespiratory bypass pathways. The Flow section illustrates sucrose transport (e.g., *SUTs*, *SWEETs*) through the phloem from companion cells to sieve elements. The Sink section highlights the regulation of sink capacity (e.g., *OsCKX2*), seed quantity/size (e.g., *OsGW2*), and plant architecture (e.g., *TaTB1*), which determine yield potential. Image elements are from BioRender (https://www.biorender.com).

Extreme climate events (heat, drought, salinization) have already reduced yields across large fractions of global cropland, making genetic gains in yield potential a key contributor to resilience under land degradation [283, 284, 285]. Yield gains can also relieve environmental pressures. For example, a 10% increase in per‐area yield may reduce land conversion needs by ∼15%, helping curb deforestation and lowering per‐unit inputs of fertilizers and pesticides [286, 287]. Mechanistically, yield reflects the interaction of genotype, environment, and management through coordinated control of yield components (grain weight, grain number, seed setting), which can be framed as optimization of the source‐flow‐sink system (Figure 4).

### 5.1 Grain Number per Panicle and Seed Setting Rate

Grain number per panicle and seed‐setting rate are pivotal determinants of crop yield, governed by complex molecular regulatory networks. In rice, OsCKX2, encoding cytokinin oxidase/dehydrogenase, regulates panicle meristem activity by degrading cytokinin (CK). Rice OsCKX2 (cytokinin oxidase/dehydrogenase) controls panicle meristem activity by CK degradation. A retrotransposon insertion in the OsCKX2 promoter lowers expression (a cis‐regulatory variant), increases CK accumulation, and was associated with ∼15%–20% more grains per panicle in japonica backgrounds [288, 289]. Similarly, the high‐yield gene GY3 enhances cytokinin biosynthesis, increasing plot yield by 7%–15% [290]. By contrast, the OsMADS17 ‐ miR156j (SDT)—IPA1 module limits spikelet production. OsMADS17 knockout increased spikelet number per panicle by 19.2% in trait phenotyping assays [291]. In wheat, *TaJAZ1*, a jasmonate signaling repressor, modulates spikelet differentiation by attenuating the JA signaling pathway. A nonsynonymous substitution (Gln→Arg) in its B‐genome allele (*TaJAZ‐B1*) enhances the number of fertile spikelets and grains per spike; near‐isogenic lines carrying this allele show a 12% increase in grain number [292]. The sucrose synthase gene TaSus1, crucial for sucrose metabolism, provides energy and carbon skeletons for spike development. Carbon supply can be another constraint: the sucrose metabolism gene *TaSus1* harbors a Val2113Leu variant in *TaSus‐A1*, and association analyses linked the Val allele with 10%–15% more grains per spike [293]. In maize, the ethylene biosynthesis gene *ZmACO2* modulates floret abortion by regulating endogenous ethylene levels. *ZmACO2* knockout reduces ethylene accumulation in female inflorescences by 40%, decreases floret abortion by 25%, and increases kernel number per ear by 13.4% [294]. The WD40 protein gene *KRN2* and its rice ortholog *OsKRN2* are conserved negative regulators of grain number through suppression of meristem activity. *KRN2* knockout in maize increases kernel rows by 2‐3 and yield by 10%, while *OsKRN2* knockout in rice raises secondary branch number by 15% and yield by 8% [295]. Not all yield‐component targets are ‘free gains’: stigma development is highly dosage‐sensitive. In maize, triple knockout of ZmSPL10/14/26 nearly abolished stigma papillae and severely impaired seed setting; likewise, knockout of the rice orthologs OsSPL5/OsSPL10 caused abnormal stigma papilla development and markedly reduced seed‐setting rate, underscoring strong pleiotropic costs in reproductive‐tissue regulators [296].

Collectively, successful increases in grain number/seed setting tend to fall into three design logics: (i) meristem activity tuning (CK, WD40 regulators), (ii) reproductive hormone balancing (ethylene/JA), and (iii) carbon supply allocation (sucrose metabolism). However, regulators acting directly in reproductive organ differentiation (e.g., SPL modules) can carry high pleiotropic risk, so promoter tuning or allele‐series approaches may be preferable to complete loss‐of‐function when breeding translation is the goal.

### 5.2 Grain Weight and Size

Grain weight/size are quantitative traits shaped by regulators of cell proliferation and expansion. In rice, map‐based cloning identified OsGW2 as a major grain‐width QTL. The low‐expression allele increases grain width by ∼12% and thousand‐grain weight by 10%–15% [14]. Mechanistically, OsGW2 encodes an E3 ubiquitin ligase that interacts with OsARF4 to repress cell‐division genes (e.g., OsCYCD3;1), and the gw2‐1 loss‐of‐function mutant shows ∼30% more hull cells with correspondingly larger/heavier grains [297, 298].

For example, in rice, OsGS3, encoding a transmembrane protein, negatively regulates grain length by inhibiting cell elongation. The gs3‐1 mutant increases grain length by 15%–20% and thousand‐grain weight by 8%–12%. OsGS3 interacts with DEP1 to coordinately regulate grain morphology and plant architecture via the brassinosteroid pathway [299, 300]. Similarly, *OsGS5*, encoding a serine carboxypeptidase, promotes hull cell division and expansion. Its high‐expression allele (*GS5‐UP*) increases grain width by 8% and thousand‐grain weight by 7%–10% [301]. OsGS5 forms a transcriptional complex with OsMADS1 to activate *OsCYCB1;1* expression [302, 303]. In wheat, an E3 ligase parallel to rice GW2‐TaGW2‐A1‐shows a clear directionality: overexpression reduces grain width and thousand‐grain weight by ∼8%–12%, whereas the TaGW2‐A1‐114bpDel loss‐of‐function allele enhances grain weight [304]. For positive regulators, the serine carboxypeptidase *TaGS5‐3A* displays favorable allelic effects, with the TaGS5‐3A‐T allele showing ∼15.2% higher thousand‐grain weight than *TaGS5‐3A‐G* [303]. Regulatory variation can also act through promoters: a TaGDSL‐7D promoter haplotype (TT/GG; HS2) strengthens *TaGT1* binding, elevates expression by ∼40%, and increases thousand‐grain weight by ∼9.8% [305]. In maize, *KRN2*, a WD40 protein, negatively regulates kernel row number and grain weight by limiting ear meristem activity. *KRN2* knockout increases kernel rows by 2‐3 and grain weight by 10%–15%, without affecting other agronomic traits [295]. The CONSTANS‐like gene *ZmCCT9* affects grain weight through photoperiod regulation and endosperm development. Its favorable allele (*ZmCCT9‐Hap2*) enhances grain weight by 12.5% and is prevalent in temperate maize germplasm (65%) [306].

Across crops, grain size/weight gains tend to follow three recurring ‘design logics’: (i) altering hull/ovary cell proliferation (E3 ligases, cyclin regulation), (ii) tuning longitudinal vs radial growth programs (GS3/GS5 modules), and (iii) leveraging cis‐regulatory variation to achieve quantitative effects with fewer pleiotropic penalties. When moving toward breeding translation, allelic series and promoter variants are often preferable to strong overexpression, which can shift resource allocation and compromise other yield components.

### 5.3 Tiller and Fruit Branch Number

Productive tiller and fruit branch number are key agronomic traits that define plant architecture, resource allocation, and yield potential. They not only influence environmental adaptability but also determine optimal planting density and management strategies in both field and horticultural crops.

Rice tillering can be tuned through nutrient‐responsive regulation and bud‐outgrowth modules. OsGATA8‐H illustrates genotype‐by‐nitrogen plasticity: under low N it enhances cytokinin biosynthesis/signaling to promote productive tillers, whereas under high N it restrains excessive tillering by suppressing tiller‐inhibitory factors, linking nitrogen‐use efficiency with tiller control [307]. Mechanistically, derepression of tiller inhibitors is another route‐RCN127 interacts with OsTB1 and OsTCP19 and promotes their 26S proteasome‐mediated degradation, which coincides with increased tiller number [87]. At the developmental program level, the DLT3‐MOC3‐MOC1 complex coordinates tiller bud development and outgrowth, supporting DLT3 as a positive regulator of productive tillering [86]. In wheat, a newly identified QTL, QPtn.sau‐4B, has been shown to enhance productive tiller number and grain yield across multiple field trials, providing valuable genetic resources for high‐yield wheat breeding [308]. In Arabidopsis, the TCP factor BRC1 functions as a hub enforcing axillary bud dormancy and integrating hormonal/environmental cues [309, 310]. Protein‐level modulation can shift this balance: TIE1, a transcriptional repressor, directly interacts with BRC1 to inhibit its activity; TIE1 overexpression increases branching but is accompanied by altered leaf morphology, and its declining expression in developing axillary buds suggests a dynamic role during bud outgrowth [311, 312]. In soybean, the Dt2 gene encodes a bHLH transcription factor that negatively regulates branching by activating GmAp1a and GmAp1d expression through direct promoter binding. Interaction with GmAgl22 and GmSoc1a further enhances this transcriptional activation, leading to the suppression of branching [313].

Across species, branch‐number engineering commonly follows two design logics: (i) modulating inhibitor hubs (e.g., TB1/TCP/BRC1‐centered repression) and (ii) tuning developmental competence of axillary buds via transcriptional complexes and hormone balance. Because excessive branching/tillering can dilute assimilates and destabilize yield components, variants with environment‐ or nutrient‐responsive plasticity (e.g., haplotypes/QTLs effective across field conditions) are often more breeding‐friendly than constitutive activation.

### 5.4 Photosynthetic Efficiency

Enhancing photosynthetic efficiency is among the most promising routes to increase crop yield [314]. Key constraints include slow photoprotective recovery, photorespiratory carbon loss, and rate limitations in electron transport [315]. Genetic engineering has therefore targeted photoprotection, electron transport, Calvin‐cycle capacity, and photorespiratory bypasses, delivering measurable gains in CO_2_ fixation, biomass, and in some cases yield [316, 317].

The xanthophyll cycle is a key photoprotective process that dissipates excess excitation energy through non‐photochemical quenching (NPQ). Overexpression of the VDE gene, encoding violaxanthin de‐epoxidase, enhances both NPQ induction and relaxation, thereby reducing photoinhibition and increasing photosynthetic efficiency. In rice, VDE overexpression accelerated NPQ induction and photosynthetic recovery under fluctuating light, increasing cumulative CO_2_ assimilation by 20%–23% and biomass by 11%–16% compared to wild‐type plants [318, 319]. Rate control in the electron transport chain often maps to the cytochrome b6f bottleneck. Increasing b6f capacity via Rieske iron‐sulfur protein overexpression improved electron transport and photosynthesis (≈10% in Setaria) and increased light‐use efficiency with biomass/yield gains in sorghum [316]. Additional support comes from enhancing electron carriers: PETE1/PETE2 (plastocyanin) overexpression increased biomass accumulation [317]. In the Calvin cycle, the enzyme Rubisco catalyzes CO_2_ fixation and directly determines photosynthetic efficiency. Expression of thermostable Rubisco activase isoforms in Arabidopsis improved Rubisco activation, leading to increased photosynthetic rate, biomass, and seed yield [317]. In rice, overexpression of the maize Rubisco activase gene enhanced Rubisco activity and photosynthetic rate under high‐temperature stress. Co‐overexpression of both Rubisco and its activase restored photosynthesis and biomass accumulation under heat conditions [319, 320]. Similarly, overexpression of sedoheptulose‐1,7‐bisphosphatase (*SBPase*)‐a key enzyme controlling the Calvin cycle flux under elevated CO_2_‐enhanced photosynthesis and biomass in tobacco, wheat, and *Arabidopsis*. Overexpression of plastid aldolase also increased photosynthetic rates, seed yield, and total biomass, whereas its suppression impaired both growth and carbon fixation [317]. Photorespiration can consume a substantial fraction of fixed carbon in C3 plants, motivating synthetic bypass strategies. In rice, three designs illustrate the principle: the GOC bypass (OsGLO3/OsOXO3/OsCATC) improved photosynthesis and grain yield; the GCGT bypass (OsGLO1 plus *E. coli* EcCAT/EcGCL/EcTSR) delivered ∼13%–27% increases in photosynthetic rate and yield; and the GMA bypass (OsGLO1 + pumpkin CmMS + rice OsAPX7) raised net photosynthesis by ∼14%–21% with grain yield gains of ∼11%–26% across transgenic lines [321]. The GCGT bypass, combining rice (OsGLO1) and E. coli enzymes (EcCAT, EcGCL, EcTSR), produced 13%–27% increases in photosynthetic rate and yield relative to wild‐type [321]. Similarly, the GMA bypass‐composed of *OsGLO1*, pumpkin *CmMS*, and rice *OsAPX7*‐raised net photosynthetic rates by 14%–21% and grain yields by 11%–26% across transgenic lines [321]. Beyond these core pathways, genes influencing chloroplast development and assimilate transport also play decisive roles in photosynthetic performance. The chloroplast‐localized TCD5 gene, encoding a putative monooxygenase, regulates photosynthetic efficiency, panicle number, and grain yield in a dose‐dependent manner. tcd5 mutants exhibited reduced panicle number (12.4%–14.6%) and grain yield (9.1%–18.4%), while TCD5 overexpression increased panicle number by up to 61.8% and yield by up to 56.5% [314]. The rice sucrose transporter OsSUT1 facilitates long‐distance assimilate transport through the apoplastic pathway, promoting efficient translocation of photosynthates to grains [322, 323]. In maize, ZmSWEET4c, encoding a sugar transporter responsible for phloem‐to‐sink sugar transfer, enhanced grain filling and yield when overexpressed [324].

Beyond ‘source’ biochemistry, phenology (flowering time and maturity duration) acts as a system‐level determinant of whether photosynthetic gains translate into stable yield across environments. By controlling seasonal fit and the ability to escape heat, drought, or frost, phenology strongly shapes yield stability and cropping‐system design. Accordingly, phenology engineering often targets (i) photoperiod/circadian modules, (ii) florigen pathways and meristem‐identity regulators, and (iii) hormonal and temperature‐responsive flowering signals. Because these nodes are highly pleiotropic, quantitative tuning (e.g., promoter/cis‐regulatory editing or weak alleles) and environment‐aware evaluation are generally preferable to strong loss‐of‐function interventions. The impact of phenology engineering should be assessed through multi‐location field trials spanning daylength and temperature gradients, with explicit reporting of yield‐component trade‐offs.

Across these examples, yield gains cluster into recurring design logics: increasing source capacity (light capture/carbon fixation), improving flow efficiency (phloem loading/unloading), and strengthening sink size/activity. Yet, effect sizes are often background‐ and environment‐dependent, and may trade off with stress tolerance or development; therefore, multi‐location field validation and component‐trait dissection are essential to avoid ‘greenhouse winners’ and to identify designs that are robust under agronomic conditions.

## 6 Genetic Engineering for Quality Improvement in Plants

**FIGURE 5:**
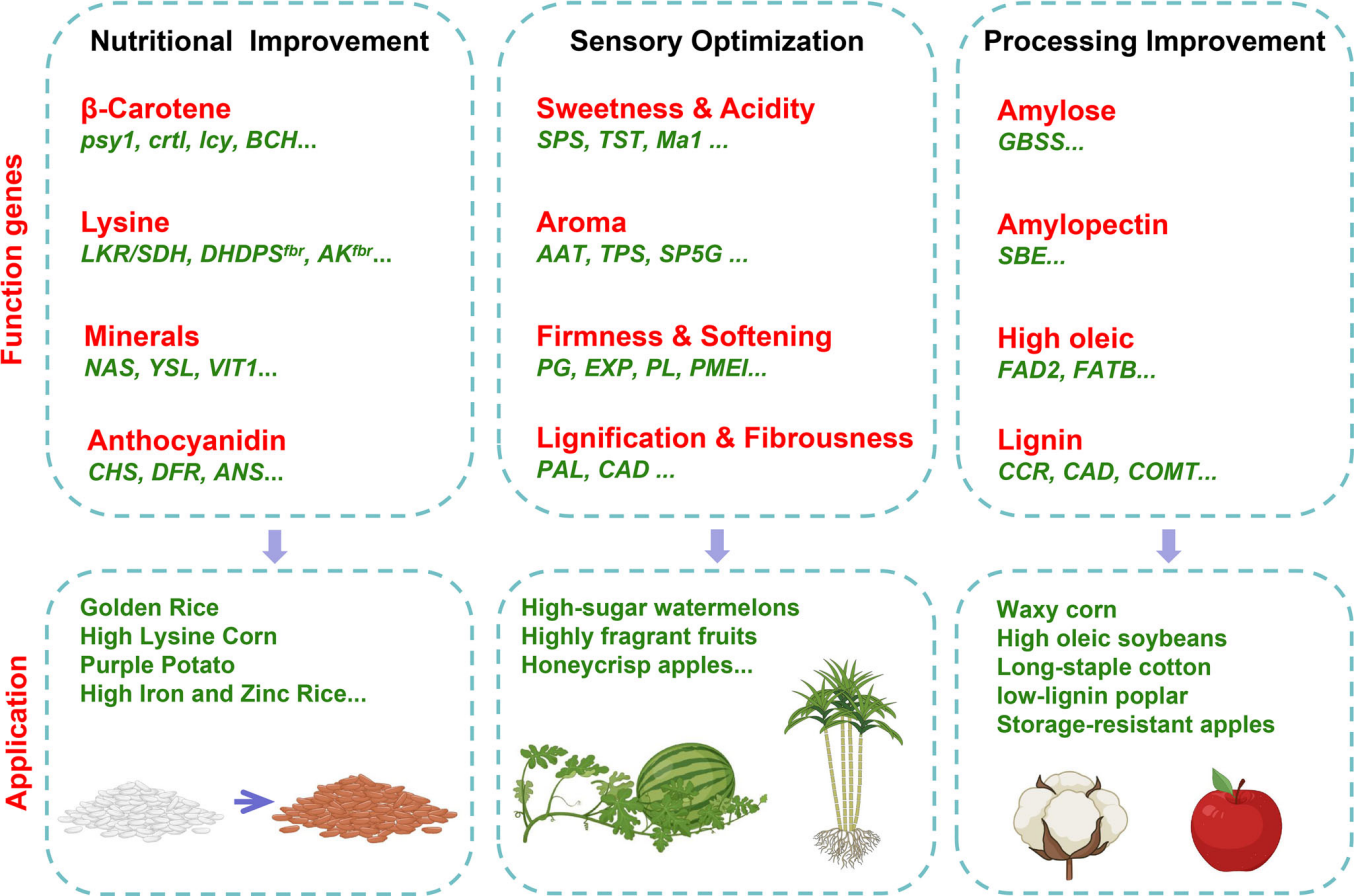
Overview of genetic engineering strategies for crop quality improvement. This schematic summarizes key molecular approaches across three domains. (1) Nutritional improvement, targeting β‐carotene, lysine, minerals, and anthocyanin enrichment through genes such as *psy1*, *DHDPS*, *NAS*, and *CHS*. (2) Sensory optimization, focusing on sweetness, aroma, and texture via the regulation of *SPS*, *AAT*, *PG*, and *PAL*. (3) Processing improvement, modifying starch, oil, and fiber composition through manipulation of *GBSS*, *SBE*, *FAD2*, and *CCR*. Representative applications include Golden Rice (provitamin A enrichment), high‐sugar watermelon, and waxy corn, showcasing the potential of metabolic engineering in quality enhancement. Image elements are from BioRender (https://www.biorender.com).

Plant genetic engineering enables targeted modification of quality‐related loci, overcoming species barriers and the randomness of conventional breeding, and thus provides an efficient route for crop quality improvement [325]. Here, we focus on three application domains‐nutritional enhancement, sensory optimization, and processing/functional‐property improvement‐that collectively shape nutritional profiles, manufacturability, and consumer acceptance. The pathway from gene discovery to industrial deployment is increasingly important for delivering nutritious, health‐promoting, and functional foods that support global food and nutrition security (Figure 5).

To make the review actionable, we organize representative cases along three questions: (i) translation stage (proof‐of‐concept vs. breeding/industry adoption), (ii) what was modified and how (transgene vs. genome editing; cis‐regulatory tuning vs. coding‐sequence changes), and (iii) trade‐offs and mitigation (growth penalties, yield dilution, and processing/sensory side effects; managed through tissue‐specific expression and pathway balancing).

### 6.1 Nutritional Quality

Natural plant‐derived foods can be nutritionally unbalanced (e.g., low essential amino acids/vitamins) and may contain anti‐nutritional factors. Genetic engineering addresses these gaps through three recurring approaches‐supplementing limiting nutrients, reducing harmful or anti‐nutritional components, and enhancing functional compounds‐to improve overall nutritional value [326]. In practice, fortification often relies on introducing or tuning key biosynthetic and transport steps to increase target nutrients in edible tissues [327].

Canonical success case is ‘Golden Rice’, which restores provitamin A (β‐carotene) biosynthesis in rice endosperm via multigene transgenic pathway reconstruction. The pathway is initiated by *Phytoene synthase 1* (PSY1), the first committed and rate‐limiting step in carotenoid biosynthesis [328]. However, PSY activity alone mainly accumulates phytoene, so crtI (a bacterial carotene desaturase from Erwinia) is introduced to drive desaturation toward lycopene, which is then converted to β‐carotene via LCY (lycopene β‐cyclase). Coordinated endosperm expression of psy1‐crtI‐lcy underpins the effectiveness of the Golden Rice strategy [22, 329]. Carotenoid fortification can also be achieved in fleshy fruits: SlLCYB2 overexpression or heterologous expression of ORANGE (OR) increases carotenoid accumulation in tomato [330, 331]. Because maize kernels are naturally low in lysine, nutritional protein quality can be improved by either reducing lysine catabolism (e.g., silencing LKR/SDH) or introducing high‐lysine storage proteins (e.g., legumin), which together can raise lysine levels by ∼2‐3 fold [332, 333, 334]. Similarly, engineering key enzymes in the lysine biosynthesis pathway has proven effective. *DHDPS* (*Dihydrodipicolinate synthase*) catalyzes the condensation of an aspartate derivative (ASA) with pyruvate to form dihydropicolinic acid‐the first committed step in lysine synthesis. Reducing feedback inhibition of *DHDPS* and *AK* (*Aspartate kinase*) by lysine in transgenic rice increased free lysine levels by 6.6‐ and 21.7‐fold, respectively, compared with wild‐type seeds [335]. Similarly, mutated feedback‐insensitive AK genes (e.g., lysC from E. coli or AK2 from maize) relieve the rate‐limiting constraints of aspartate phosphorylation, enhancing flux through the entire aspartate‐family amino acid pathway and supplying abundant precursors for lysine biosynthesis [336, 337].

Iron and zinc deficiencies remain widespread micronutrient malnutrition challenges. Engineering solutions typically target an uptake‐chelation‐translocation chain to increase delivery into edible tissues. At the uptake step, IRT1 is a high‐affinity root iron transporter induced under Fe deficiency, and its elevated expression can expand the soil‐to‐root acquisition pool [338, 339]. For long‐distance movement, NAS increases nicotianamine (NA) production, strengthening Fe/Zn chelation and mobilization toward developing seeds to overcome internal transport bottlenecks [340, 341]. Finally, YSL transporters move metal‐NA complexes through vascular loading/unloading, and higher YSL activity improves Fe/Zn translocation into endosperm tissues [342, 343]. Because micronutrient accumulation can be limited by source availability and may interact with growth or stress responses, strategies that combine uptake, transport, and tissue‐specific storage often translate more robustly than single‐gene interventions.

### 6.2 Sensory Quality

Plant sensory quality is largely shaped by three chemical/structural layers: soluble carbohydrates (sweetness), volatile organic compounds (aroma), and cell‐wall remodeling (texture/firmness). Genetic engineering can tune key biosynthetic, transport, and degradation nodes within these layers to improve desirable sensory attributes. Sweetness is largely determined by the concentration of soluble sugars such as sucrose, fructose, and glucose, along with certain amino acids. Enhancing sugar accumulation and transport represents a key strategy for sweetness improvement. In tomatoes, sweetness is mainly dependent on sucrose and glucose content, and sucrose phosphate synthase (SPS)‐a key enzyme in sucrose biosynthesis‐plays a central role. Accordingly, elevating sucrose biosynthetic capacity through SPS overexpression increases sucrose accumulation and perceived sweetness in tomato [344, 345]. Sugar storage is also controlled by vacuolar sequestration: tonoplast sugar transporters (TSTs) move sugars from the cytosol into the vacuole, shaping intracellular sugar partitioning. Increasing this sink capacity‐e.g., via TST overexpression‐enhances vacuolar import, reduces cytosolic feedback constraints, and supports sustained sugar accumulation [346].

Aroma is composed of hundreds of volatile organic compounds (VOCs), mainly esters, terpenes, aldehydes, alcohols, and phenols. Among aroma VOCs, esters are major contributors to ‘fruity’ notes, and alcohol acyltransferases (AATs) catalyze their formation from alcohols and acyl‐CoAs [347, 348]. Increasing AAT activity (e.g., by overexpression) boosts the abundance/diversity of ester volatiles and intensifies fruity aroma profiles [349]. Terpene synthases (TPSs) catalyze the formation of diverse terpene volatiles‐such as limonene (lemon), linalool (floral scent), and myrcene‐using universal precursors (GPP, FPP, GGPP). Overexpressing specific *TPS* genes in target crops can introduce or enhance distinct floral, woody, or citrus aromas [350, 351]. Visual and textural qualities can also be optimized through genetic modification. For visual appeal, color traits can be engineered by activating pigment pathways. Potatoes naturally lack anthocyanins; introducing grape anthocyanin biosynthetic genes (e.g., CHS and DFR) enables transgenic tubers to accumulate anthocyanins, producing purple skin/flesh with added antioxidant value [352, 353, 354, 355, 356].

Fruit texture is largely determined by the structure and composition of the cell wall, which undergoes dynamic remodeling during ripening. Polygalacturonase (PG) is one of the main enzymes responsible for pectin degradation, hydrolyzing α‐(1,4)‐glycosidic bonds and causing cell wall loosening and softening. Suppressing *PG* expression via antisense RNA or RNAi effectively delays fruit softening and extends shelf life‐a classical example of postharvest improvement through genetic engineering [357, 358]. Expansin (EXP) proteins, though not directly hydrolytic, loosen the cell wall by disrupting hydrogen bonds between pectins and cellulose microfibrils, promoting cell enlargement and reducing tissue firmness during ripening. Inhibiting Expansin expression helps maintain cell wall integrity and enhances fruit firmness. For example, antisense suppression of LeEXP1 in tomato markedly reduced softening [359, 360]. Additionally, pectin methylesterase (PME) demethylates pectin, creating sites for PG activity, while its inhibitor (PMEI) prevents this process. Overexpression of PMEI suppresses PME activity, thereby maintaining pectin esterification and delaying fruit softening [361].

Across sensory traits, successful interventions typically target either flux/partitioning control (e.g., SPS/TST for sugars) or key terminal enzymes that define dominant aroma/texture outputs (e.g., AAT/TPS; PG/EXP/PME). Because sensory gains can trade off with growth, ripening kinetics, or storage physiology, strategies that tune expression spatially/temporally often translate more reliably than constitutive overexpression.

### 6.3 Processing Quality

Processing quality‐including starch functionality, oil composition/stability, and fiber/lignin properties‐directly determines the performance of crops as raw materials for food, textile, and bioenergy industries. Genetic engineering can reprogram key metabolic nodes to align end‐use traits with industrial specifications.

Starch processing quality is strongly influenced by the amylose/amylopectin ratio and gelatinization behavior. In maize (≈25% amylose), reducing amylose synthesis by GBSSI silencing‐a key enzyme for amylose chain elongation‐can shift starch composition and functionality [362, 363]. Precision tuning has also been demonstrated in rice: CRISPR/Cas9 co‐editing of SSIIa and Wx (Waxy) adjusted amylose content and gel consistency, generating germplasm with improved cooking, eating, and processing properties [364, 365]. Oil processing quality depends on fatty‐acid saturation, which affects both nutrition and oxidative stability. A primary engineering lever is FAD2, the ER desaturase converting oleic acid (C18:1) to linoleic acid (C18:2) [366]; reducing FAD2 activity via RNAi knockdown or CRISPR/Cas9 knockout can yield high‐oleic oils (>80%) with improved stability and reduced need for hydrogenation [367, 368, 369]. A complementary lever targets saturated‐fat formation: plastid FATB terminates saturated acyl‐ACP chains, and its suppression redirects flux toward unsaturated fatty acids, lowering saturated fat content [252, 370]. Fiber‐related traits such as cotton fiber length, strength, and wood lignin content are also critical for industrial applications. Knockout of Phosphatidylethanolamine‐Binding Protein (GhPEBP) in cotton significantly improved fiber length and tensile strength [371]. In poplar, simultaneous editing of CCR (Cinnamoyl‐CoA Reductase) and Cinnamyl Alcohol Dehydrogenase (CAD) in the lignin biosynthetic pathway reduced lignin content and increased the cellulose‐to‐lignin ratio, enhancing pulping efficiency [372].

Ethylene (C_2_H_4_), is a central regulator of fruit ripening and senescence, and thus influences postharvest and processing quality. Its biosynthesis is controlled by ACS (SAM to ACC; rate‐limiting) and ACO (ACC to ethylene) [373, 374]. Rather than creating completely ethylene‐deficient mutants, regulatory tuning can preserve quality while extending shelf life: editing SlERF.F12 produced tomato lines that ripened more slowly yet maintained normal coloration [375]. Consistent with this principle, natural ACS allelic variants with reduced enzyme activity have been associated with extended storage capacity in apple cultivars [376, 377].

Across processing traits, successful designs often either (i) shift polymer composition (starch branching/amylose; lignin monomer flux) or (ii) re‐balance lipid saturation to optimize stability and health attributes. Because these modifications can affect growth, storage physiology, or sensory outcomes, pathway‐level tuning and end‐use testing are essential to avoid improvements in one processing metric at the expense of overall quality.

## 7 Prospects

To transform the future outlook from a list of emerging topics into a coherent roadmap, this section is organized around four integrative questions: (i) What are the current bottlenecks in plant genetic engineering and where are the most promising breakthroughs likely to emerge? (ii) How can novel biotechnologies be integrated synergistically with conventional breeding pipelines rather than viewed as competitors? (iii) How should risks and opportunities of biotech crops be evaluated with respect to human health and environmental sustainability? (iv) At a macro level, how can genetic engineering contribute to climate adaptation and mitigation, from molecular mechanisms to agroecosystem outcomes? We use these questions to connect enabling technologies (editing and transformation), genetic resources (wild relatives), regulatory layers (epigenetic control), and translation requirements (field validation) into a unified design‐build‐test‐learn framework.

### 7.1 Emerging Enabling Technologies for Next‐Generation Crop Engineering

Current breeding‐by‐engineering is limited less by the availability of targets than by three practical bottlenecks: (i) creating quantitative, low‐pleiotropy alleles for complex traits, (ii) delivering reagents efficiently across diverse genotypes without tissue‐culture penalties, and (iii) overcoming recombination constraints and linkage drag at the chromosome scale. The enabling technologies discussed below map directly onto these bottlenecks and collectively shorten the design‐build‐test‐learn loop.

To address the bottleneck of engineering complex traits with manageable pleiotropy, cis‐regulatory motif and promoter engineering provides a scalable route to generate quantitative allelic series for breeding, enabling fine‐tuning of gene expression with reduced penalties compared with complete knockouts. Promoter editing has been demonstrated to create beneficial quantitative variation in crops, including *cis*‐regulatory alleles in tomato and yield‐related quantitative promoter alleles in maize [378]. Moreover, the increasing availability of open‐chromatin and single‐molecule regulatory maps can substantially improve target prioritization for noncoding edits: Assay for Transposase‐Accessible Chromatin using sequencing (ATAC‐seq) supports the identification of candidate functional *cis*‐regulatory elements, whereas chromatin fiber sequencing (Fiber‐seq) provides long‐read, single‐molecule accessibility architectures that help resolve complex regulatory regions [379, 380]. Integrating these multi‐omic regulatory datasets with machine learning (ML) models for variant‐effect prediction is expected to reduce empirical screening burden and accelerate the design‐build‐test cycle for regulatory engineering.

Against this backdrop, emerging transformation routes‐including organelle (mitochondrial/chloroplast) transformation and leaf‐based transformation/regeneration platforms‐could expand the range of editable germplasm and reduce turnaround time [7, 28]. Because tissue culture is often genotype dependent, slow, and prone to somaclonal variation, developmental‐regulator‐assisted regeneration and de novo meristem induction offer a practical path to bypass or simplify culture steps, accelerating reagent delivery and broadening editable germplasm. Pangenomics contributes to crop genetic improvement primarily by capturing genetic variation in wild progenitors that is associated with traits such as disease resistance, tolerance to abiotic stresses, and environmental adaptation. This provides a theoretical foundation for developing crop varieties with enhanced resistance and resilience, and helps guide rational, science‐based breeding. With advances in sequencing technologies and the rapid reduction in sequencing costs, pangenomes have been constructed for an increasing number of species, which is of great significance for in‐depth studies on functional gene identification, domestication, and related topics [381].

A third bottleneck is linkage drag and constrained recombination, and beyond nucleotide‐scale edits, CRISPR‐based chromosome engineering (programmable inversions/translocations and other structural variants) provides a route to reshape linkage blocks and recombination landscapes for breeding [46]. By breaking unfavorable linkage drag and enabling targeted reshaping of chromosomal architecture, these approaches can complement allele‐level editing and facilitate trait stacking at the chromosome scale. Taken together, these three technology layers‐quantitative regulatory editing, culture‐light delivery/regeneration, and chromosome‐scale restructuring‐are complementary: they expand what can be built, in which germplasm it can be built, and how efficiently desirable allele combinations can be assembled for breeding translation.

### 7.2 Multigene Co‐Transformation and Simultaneous Editing

Complex agronomic outcomes (architecture, stress resilience, yield, and quality) are rarely optimized by single‐gene changes. Rather than competing with conventional breeding, multigene engineering and simultaneous editing are most powerful when embedded within a design‐build‐test‐learn (DBTL) pipeline: engineered alleles/modules are created and stacked ('build'), then conventional breeding provides background optimization, segregation of transgene footprints when needed, and multi‐environment evaluation ('test'), with data feeding back into the next design cycle. In this context, multigene co‐transformation and multiplex editing expand what can be built, while breeding pipelines determine what reliably translates [382, 383].

Multigene co‐transformation aims to achieve stable integration and coordinated expression of multiple genes, enabling pathway reconstruction or functional‐module transfer. Compared with early ‘random stacking’ approaches that often produced unpredictable insertion contexts and imbalanced expression [384], current strategies increasingly rely on structured assembly and expression‐balancing designs. For example, early Ti‐plasmid co‐transformation of Hsp70 and P5CS in rice was sometimes associated with insertion‐related penalties and reduced seed setting, underscoring the need for controlled integration and expression balancing [385, 386]. Optimization of multigene expression systems has since expanded from the introduction of stress‐resistance genes to the reconstruction of key metabolic pathways. Given that nitrogen is a fundamental nutrient for plant growth, conferring autonomous nitrogen fixation capacity represents a transformative goal for sustainable agriculture. Through approaches such as multigene assembly, single transformation, co‐transformation, and hybridization, thirteen nitrogen fixation genes (∼40 kb) from two nitrogen‐fixing bacteria were successfully introduced into rice. Genome resequencing confirmed their integration at two loci on chromosome 1, and formation of the NifDK tetrameric nitrogenase complex was verified‐offering a crucial reference for achieving “autonomous nitrogen fixation” in graminaceous crops [382]. This work demonstrates how multigene co‐transformation, guided by synthetic biology principles, can transcend interspecies metabolic barriers and facilitate the transfer of complex physiological functions across taxa. Moreover, vector system innovation continues to expand species applicability. For instance, polycistronic vectors employing “2A peptides” enabled equimolar expression of four genes in *Arabidopsis* [387], while binary bacterial artificial chromosome (BIBAC) vectors enhanced the efficiency of introducing large, multigene DNA fragments into maize [388, 389]. The evolution of CRISPR‐Cas systems has provided a powerful framework for simultaneous multigene editing. Research focus has shifted from single‐gene modifications toward systematic manipulation of entire gene networks, driven by continuous advances in editing precision and multiplexing capability. In crop improvement, the benefits of multigene editing are particularly evident [390, 391]. For example, in salt‐tolerant rice breeding, 13 trait‐related genes were simultaneously targeted in the “Sea Rice 86” variety. The resulting SR86M line displayed mutations in all 13 loci, with nine confirmed desired edits and seven traits substantially improved‐shorter stature, optimized plant architecture, slender grains, increased grain number, reduced photoperiod sensitivity, enhanced aroma, and improved nitrogen‐use efficiency. These modifications rendered the line more suitable for large‐scale cultivation while maintaining the original salt tolerance, highlighting multigene editing as an efficient platform for rapid trait stacking and novel germplasm creation [392].

Recent innovations have also refined plant prime editing systems through the redesign of pegRNA scaffolds, engineering of reverse transcriptase domains, and optimization of fusion protein configurations. This upgraded system enables precise simultaneous editing of 2‐8 genes in wheat, targeting multiple agronomic traits such as disease resistance, herbicide tolerance, grain quality, and yield. Moreover, the PrimeRoot editor now supports precise insertion of large DNA fragments (>10 kb) into plant genomes. In rice, this tool enabled site‐specific integration of promoter sequences into the 5′UTR of target genes, accelerating the development of disease‐resistant cultivars [393]. Despite significant progress, multigene transformation and editing technologies still face critical challenges, including ethical considerations, high technical costs, and limited foundational data. For instance, potential environmental impacts of gene‐edited crops necessitate long‐term ecological monitoring [394]. Key future directions include: (1) Enhancing editing efficiency and reducing off‐target effects: While CRISPR systems are highly effective, multiplex editing amplifies the risk of unintended mutations. High‐fidelity Cas variants (e.g., HiFi Cas9) and optimized delivery approaches such as ribonucleoprotein (RNP) complexes are essential for minimizing these risks [395]. (2) Preventing chromosomal structural variations: Concurrent double‐strand breaks at multiple loci can cause translocations or deletions. Using base or prime editors can mitigate these issues [396]. (3) Rational design of complex trait networks: Identifying optimal editing combinations from large genomic datasets requires integration of systems biology, artificial intelligence, and predictive modeling to achieve “genome‐designed breeding.” Interdisciplinary collaboration‐merging molecular biology, computational modeling, and synthetic biology‐will refine gene network predictions and accelerate the rational engineering of crop traits. With continued improvements in construct design, multiplex precision, and large‐fragment writing, multigene engineering is likely to become a routine ‘build’ layer for climate‐resilience and yield‐stability breeding, especially when coupled to conventional selection and multi‐environment testing [397, 398].

### 7.3 Reshaping Future Crops Through the Genetic Wealth of Wild Relatives

In a DBTL breeding pipeline, wild relatives primarily contribute an ‘allele‐supply’ layer: they expand the design space with stress‐, yield‐, and quality‐enhancing variants, while modern engineering tools enable rapid writing/stacking of these alleles and conventional breeding ensures background optimization and field stability. Below, we summarize where wild genetic resources add the most value, the key bottlenecks (incompatibility and linkage drag), and the emerging gene‐centric solutions.

Modern agriculture's reliance on a narrow set of elite cultivars has reduced genetic diversity and weakened resilience to abiotic and biotic stresses. Crop wild relatives (CWRs) retain extensive allelic and epigenetic variation shaped by natural selection, providing essential raw material for gene‐centric crop design [399]. Harnessing this diversity‐while overcoming practical utilization bottlenecks‐will be crucial for sustainable intensification and food security [400].

The unique advantages of wild germplasm are reflected in three key dimensions. First, wild relatives exhibit remarkable tolerance to abiotic stresses. Many species thrive under drought, salinity, high temperature, and nutrient‐poor conditions, having evolved distinct physiological and molecular mechanisms of adaptation. For example, wild tomato (*Solanum pennellii*) demonstrates exceptional water‐use efficiency under drought stress, while wild wheat (*Aegilops tauschii*) and wild barley (*Hordeum spontaneum*) display strong tolerance to saline‐alkali soils [401, 402]. Similarly, the D‐genome wild cotton species diverged from cultivated counterparts approximately 0.5‐2 million years ago [403, 404] and evolved resilience to extreme drought, cold, and pest pressure in harsh, non‐domesticated environments. Gossypium thurberi, native to the frost‐prone Sonoran Desert, has evolved tolerance to short‐term freezing at −6°C [405]. These complex adaptive traits, typically governed by multiple quantitative trait loci (QTLs), exhibit far greater genetic complexity than that found in modern cultivars. QTL mapping and gene cloning can pinpoint the causal genes or regulatory elements behind these adaptations, enabling either introgression or precise rewriting/editing of elite backgrounds to improve climate resilience [406]. Wild relatives harbor abundant resistance genes against pathogens and pests, reflecting long‐term co‐evolution with biotic threats. These include numerous R genes and defense regulators that confer broad‐spectrum and durable resistance. A classical example is the bacterial blight resistance gene Xa21, discovered in wild rice (Oryza rufipogon), which encodes a receptor kinase effective against multiple Xanthomonas oryzae races [407, 408]. Similarly, the *Pi9* gene from wild rice, encoding an NBS‐LRR protein, provides wide‐spectrum resistance to rice blast [409], while Rpi‐blb2 from wild potato offers potent resistance to late blight. Because many of these resistance genes remain novel to current pathogen populations, they confer durable and long‐lasting protection compared with resistance genes already eroded by pathogen adaptation [410, 411]. Finally, wild relatives offer immense potential for improving crop yield and nutritional quality. Wild germplasm is not solely associated with stress resistance‐it also retains beneficial alleles lost during domestication, including those linked to higher levels of vitamins, antioxidants (e.g., anthocyanins, carotenoids), favorable fatty acid profiles, and micronutrient accumulation [412]. For instance, wild tomatoes contain several‐fold higher concentrations of sugars, vitamin C, and antioxidants than cultivated varieties [413, 414]. By reintroducing such alleles through “re‐domestication” or biofortification, breeders can create “designer crops” that integrate high yield with superior nutritional profiles [413]. Some wild species also carry yield‐related traits, such as enhanced tillering or greater grain number per panicle, offering genetic leverage to surpass the yield plateau of modern cultivars.

Despite their enormous potential, the direct utilization of wild germplasm faces substantial challenges. Hybrid incompatibility due to reproductive isolation often leads to sterile or nonviable hybrids [415, 416]. Linkage drag remains a major constraint‐beneficial genes are frequently tightly linked with undesirable traits such as seed shattering, dormancy, or low yield, necessitating extensive backcrossing and selection cycles to isolate desired alleles [417, 418]. Additionally, recessive trait expression can obscure desirable phenotypes in early generations, complicating trait fixation.

**FIGURE 6:**
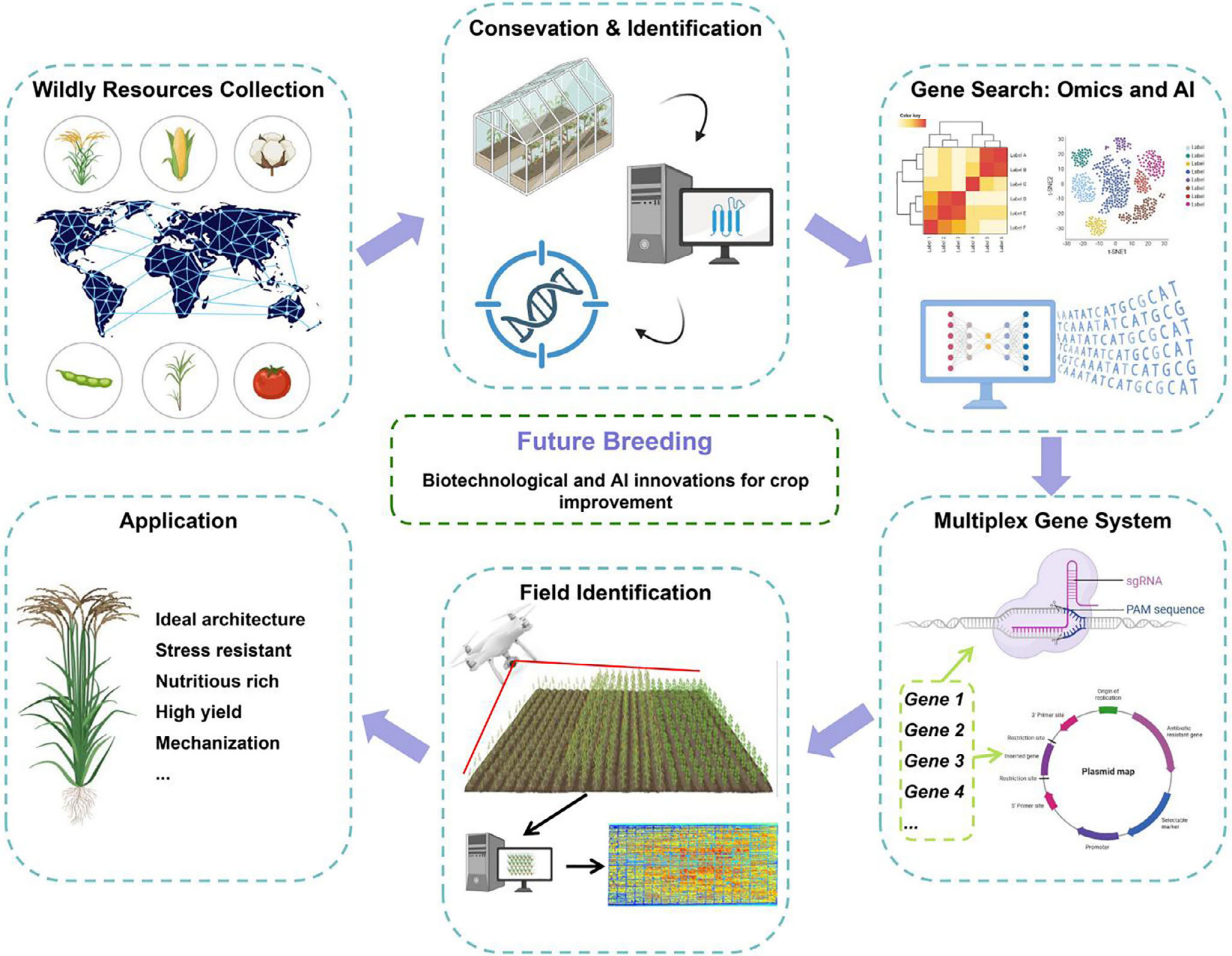
Integrative framework for harnessing wild genetic resources and advanced technologies in future crop breeding. The schematic outlines a comprehensive breeding pipeline beginning with the collection and conservation of wild genetic resources possessing advantageous traits (e.g., superior architecture, stress tolerance). Field‐based phenotypic evaluation combined with omics technologies and AI‐assisted gene discovery enables the identification of key target genes. A multiplex gene editing platform utilizing sgRNA and PAM sequences facilitates precise, multi‐gene manipulation. Together, these strategies advance the integration of biotechnology and artificial intelligence in accelerating the creation of resilient, high‐yield crop varieties for sustainable agriculture. Image elements are from BioRender (https://www.biorender.com).

Looking ahead, the integration of wild resources into crop breeding is shifting from a slow, empirical process to a data‐driven, gene‐centric, and engineering‐based paradigm. Practically, this paradigm shifts CWR utilization from slow backcrossing to a pipeline in which candidate alleles are nominated by multi‐omics, validated functionally, and then stacked or rewritten into elite lines using multiplex editing or large‐fragment integration, followed by conventional selection and multi‐environment testing. By harnessing multi‐omics datasets‐including pangenomic, transcriptomic, and epigenomic resources‐researchers can systematically identify and catalog beneficial alleles hidden within wild gene pools. Using advanced gene‐editing platforms, these elite alleles and regulatory modules can be precisely integrated into the genomic backgrounds of modern cultivars, enabling the rational design of “dream crops” that unite high yield, resilience, and superior quality. This transformative approach represents not only the restructuring of crop genomes but also the reinforcement of agricultural ecological resilience and long‐term sustainability‐offering a strategic path toward global food security in the face of intensifying environmental challenges. An integrative framework for leveraging wild genetic resources together with advanced engineering toolkits is summarized in Figure 6. Because these alleles and modules are deployed into modern agroecosystems, their success ultimately depends on risk‐benefit evaluation and field durability, as well as climate‐smart outcomes at system scale.

### 7.4 Enhancing the Precision and Stability of Epigenetic Regulation

Epigenome engineering is best viewed as a complementary layer to DNA‐sequence editing: it offers reversible, dosage‐like regulation that may reduce pleiotropic penalties for stress and developmental traits. Its translational value will depend on whether targeted marks can be made predictable, persistent, and heritable within breeding pipelines.

Gene expression is governed not only by DNA sequence but also by epigenetic layers such as DNA methylation, histone modifications, and non‐coding RNAs [419]. Some stress‐associated marks can persist and, in limited cases, be inherited across generations, motivating the idea of epigenetic ‘memory’ for climate adaptation [420]. However, applying epigenetic regulation to stress‐resistance breeding remains challenging, primarily due to its limited controllability and predictability. A major constraint lies in the incomplete understanding of how specific epigenetic variations stably and selectively regulate key stress‐responsive genes. Although genome‐wide epigenetic changes can be observed under stress conditions, achieving precise modification of methylation or histone patterns at target gene promoters‐such as *NPR1* or *WRKY* family transcription factors‐remains technically difficult. CRISPR‐based epigenetic editors (e.g., dCas9‐DNMT3A or dCas9‐TET1) are still being optimized in plants, with currently suboptimal efficiency, specificity, and persistence [421, 422]. Moreover, the heritability and stability of epigenetic marks across generations remain largely unresolved. During reproduction, extensive epigenetic reprogramming resets most marks; only a subset of loci behaves as stable epialleles, and the determinants of this stability remain incompletely understood [423]. For stress‐resistant crop lines generated through epigenetic editing, ensuring the stable transgenerational inheritance of these beneficial marks is essential‐otherwise, their effects may diminish within a few generations, undermining breeding value. This highlights the need to clarify germline epigenetic reprogramming and to identify loci or architectures that resist resetting and support stable inheritance.

Despite these obstacles, epigenetic regulation offers unprecedented opportunities for crop improvement. Unlike transgenic approaches, reactivating silenced endogenous resistance genes through targeted epigenetic editing could yield enhanced stress tolerance without introducing foreign DNA, potentially easing public and regulatory concerns [419]. Furthermore, leveraging environmentally induced epigenetic “priming” may enable crops to adopt a flexible, energy‐efficient “preparedness state,” maintaining low metabolic costs under normal conditions but mounting rapid, robust defenses when challenged by stress. Moving from proof‐of‐concept to breeding utility will require (i) improved locus‐specific writing/erasing tools with measurable persistence, (ii) experimental designs that quantify costs/benefits under field‐like stress regimes, and (iii) strategies to secure inheritance (or, alternatively, to deploy epigenetic states as controllable, non‐heritable ‘priming’ inputs within managed production systems).

### 7.5 Risk‐Opportunity Analysis and Field Durability Evaluation

Modern biotechnologies such as CRISPR‐Cas9 and transgenic engineering have accelerated the discovery and cloning of resistance genes, yet translation from laboratory success to high‐performing, field‐adapted cultivars remains a major bottleneck [424, 425]. Performance in controlled environments often overestimates real‐world efficacy because field conditions introduce heterogeneous biotic pressures, fluctuating climate, and genotype‐by‐environment interactions; therefore, field durability and multi‐environment validation should be treated as the primary filters for deployment. To evaluate biotech crops responsibly, we propose a practical risk‐opportunity matrix that considers four coupled dimensions: (i) human health and food safety (e.g., compositional equivalence, allergenicity/toxicology signals), (ii) environmental outcomes (gene flow, non‐target organisms, soil/rhizosphere effects, resistance evolution in pests/pathogens), (iii) genomic integrity (off‐target edits and structural variants, especially under multiplex editing), and (iv) agronomic durability (yield stability, trade‐offs, and multi‐year, multi‐location performance). This framework links molecular design choices to real‐world outcomes and clarifies what should be measured before large‐scale deployment.

In conclusion, realizing the full potential of genetic and epigenetic engineering for crop improvement demands a shift from single‐gene functional studies toward holistic, field‐oriented evaluations. Future research should systematically test resistance efficacy under complex environmental conditions, integrate multigene stacking strategies for durability, and refine the controllability and heritability of epigenetic regulation. By uniting the power of genomics, epigenomics, synthetic biology, and advanced breeding, it will be possible to create next‐generation “future crops” that combine high yield, superior quality, broad‐spectrum resistance, and robust environmental adaptability‐unlocking the full promise of molecular breeding for sustainable agriculture. In the next decade, plant genetic engineering will be shaped by breakthroughs that remove delivery/regeneration bottlenecks and enable quantitative, multiplex, and chromosomal‐scale design. Its impact will be maximized when integrated with conventional breeding for background optimization and multi‐environment testing. Responsible deployment requires transparent risk‐benefit evaluation linked to field durability, and climate‐smart outcomes will depend on stacking resilience traits and resource‐use innovations that translate from molecular mechanisms to agroecosystem performance.

## Authors' contributions

Conceptualization, WP and QY; writing original draft preparation, WP; Data Curation, WD and WW; Review and editing, WP, WQ and QY; funding acquisition, QY. All authors have read and agreed to the published version of the manuscript. All authors read and approved the final manuscript.

## Funding

This work was funded by Chinese Academy of Tropical Agricultural Sciences for Science and Technology Innovation Team of National Tropical Agricultural Science Center (CATASCXTD202402), Guangxi Science and Technology Project (Agricultural and Rural Field) (GUIKENONG AB24153007 and GUIKENONG AB24153001), Project of State Key Laboratory of Tropical Crop Breeding (NKLTCBCXTD24, NKLTCBCXTD38, NKLTCB‐HZ04, SKLTCLHZD202502, SKLTCBQN202514 and SKLTCBZRJJ202501), Central Public‐interest Scientific Institution Basal Research Fund (1630052025013 and 1630052025021), and China Agriculture Research System of MOF and MARA (CARS‐17).

## Conflicts of Interest

The authors declare no conflicts of interest.

## Informed Consent Statement

Not applicable.

## Data Availability declaration

The authors confirm that the data supporting the findings of this study are available within the article and its supplementary materials.

## Supporting information




**Supporting File**: ADVS74937‐sup‐0001‐TableS1.xls
